# Silent peripheral neuropathy determined by high-resolution ultrasound among contacts of patients with Hansen's disease

**DOI:** 10.3389/fmed.2022.1059448

**Published:** 2023-01-17

**Authors:** Glauber Voltan, Wilson Marques-Júnior, Jaci Maria Santana, Claudia Maria Lincoln Silva, Marcel Nani Leite, Natália Aparecida De Paula, Fred Bernardes Filho, Josafá Gonçalves Barreto, Moises Batista Da Silva, Guilherme Conde, Claudio Guedes Salgado, Marco Andrey Cipriani Frade

**Affiliations:** ^1^Healing and Hansen's Disease Laboratory, Ribeirão Preto Medical School, University of São Paulo, Ribeirão Preto, São Paulo, Brazil; ^2^National Referral Center for Sanitary Dermatology and Hansen's Disease, Dermatology Division, Internal Medicine Department, Ribeirão Preto Medical School, University of São Paulo, Ribeirão Preto, São Paulo, Brazil; ^3^Division of Neuromuscular Disorders, Department of Neurosciences and Behavioral Sciences, Ribeirão Preto Medical School, University of São Paulo, Ribeirão Preto, São Paulo, Brazil; ^4^Dermato-Immunology Laboratory, Institute of Biological Sciences, Federal University of Pará, Marituba, Brazil; ^5^Decision Support Laboratory, Federal University of Pará West, Santarem, Brazil

**Keywords:** leprosy, Hansen's disease, household contacts, neuropathy, high-resolution ultrasound, cross-sectional area (CSA), multiple mononeuropathy

## Abstract

**Introduction:**

Hansen's disease (HD) primarily infects peripheral nerves, with patients without HD being free of peripheral nerve damage. Household contacts (HHCs) of patients with HD are at a 5–10 times higher risk of HD than the general population. Neural thickening is one of the three cardinal signs that define a case of HD according to WHO guidelines, exclusively considering palpation examination that is subjective and may not detect the condition in the earliest cases even when performed by well-trained professionals. High-resolution ultrasound (HRUS) can evaluate most peripheral nerves, a validated technique with good reproducibility allowing detailed and accurate examination.

**Objective:**

This study aimed to use the peripheral nerve HRUS test according to the HD protocol as a diagnostic method for neuropathy comparing HHCs with healthy volunteers (HVs) and patients with HD.

**Methods:**

In municipalities from 14 different areas of Brazil we selected at random 83 HHC of MB-patients to be submitted to peripheral nerve ultrasound and compared to 49 HVs and 176 HD-patients.

**Results:**

Household contacts assessed by HRUS showed higher median and mean absolute peripheral nerve cross-sectional area (CSA) values and greater asymmetries (ΔCSA) compared to HVs at the same points. Median and mean absolute peripheral nerve CSA values were higher in patients with HD compared to HCCs at almost all points, while ΔCSA values were equal at all points. Mean ± SD focality (ΔTpT) values for HHCs and patients with HD, respectively, were 2.7 ± 2.2/2.6 ± 2.2 for the median nerve, 2.9 ± 2.7/3.3 ± 2.9 for the common fibular nerve (*p* > 0.05), and 1.3 ± 1.3/2.2 ± 3.9 for the ulnar nerve (*p* < 0.0001).

**Discussion:**

Considering HRUS findings for HHCs, asymmetric multiple mononeuropathy signs (thickening or asymmetry) in at least 20% of the nerves evaluated could already indicates evidence of HD neuropathy. Thus, if more nerve points are assessed in HHCs (14 instead of 10), the contacts become more like patients with HD according to nerve thickening determined by HRUS, which should be a cutting-edge tool for an early diagnosis of leprosy cases.

## Introduction

Hansen's disease (HD), one of the oldest chronic infectious diseases affecting humans, whose etiologic agent is *Mycobacterium leprae* (*M. leprae*) and *Mycobacterium lepromatosis*, an obligate intracellular pathogen with tropism for macrophages and Schwann cells, primarily infects peripheral nerves and involves the skin and other tissues (1–3). HD is a current and challenging disease still representing a public health problem in developing countries such as Brazil, which ranks second in the world in the number of new cases per year, with more than twenty thousand new cases per year (4, 5). HD has no primary prevention, which means that there is no specific vaccine against *M. leprae*, and diagnostic and prognostic tests are not feasible or well-established in clinical routine (3). The incubation period of HD is variable, ranging from 6 months to more than 20 years, with an average period of 2–4 years, due to its very slow growth (6).

The predominance of multibacillary (MB) cases with nerve impairment indicate a late diagnosis and underscores the ineffectiveness of epidemiological control in many countries (7). Moreover, new cases not only involving high functional impairment but also affecting children reflect the failure of early HD detection and indicate continued transmission (8). People with untreated HD are generally considered to be the main source of transmission; however, because of the complex relationships between genetic, immunological, and environmental factors, most infected contacts will not develop HD, although recent studies have reported that they may be healthy carriers and transmit *M. leprae* to susceptible individuals (9–13). Some authors have demonstrated the presence of viable *M. leprae* strains in skin smear samples from patients as well as in environmental samples obtained from around their homes, revealing that the occurrence of new cases among people without previous contact with those with untreated HD may be due to other undisclosed sources of infection such as water, soil, and animals (14–17).

Contacts of MB patients diagnosed with HD are at 5 to 10 times higher risk of HD than the general population (1, 18, 19). Contact with patients with HD is the main determinant of the incidence of HD (10, 13, 19–29), and the type of contact is not limited to family relationships (18).

There are no patients with HD without peripheral nerve damage, but the exact mechanism underlying the condition is still unknown (30). Scollard (30) suggested that neuropathy may occur partly by the invasion of Schwann cells from the outside to inside, and Graham Weddell observed that HD-related damage occurs at places where there is movement, such as the wrist, elbow, knee, and ankle. Such movements lead to micro-trauma to which the body responds by sending repair cells, including macrophages. For these cells to get into the endoneurium, where the micro-trauma is located, the endothelial cells of the blood vessels in the endoneurium will express adhesion molecules (30).

In a study assessing the neuropathy occurring in HD, Defaria (31) concluded that the condition is a mixed primary peripheral polyneuropathy involving motor and sensory fibers whose earliest neural lesion appears by asymmetric axonal neuropathy and as diffuse demyelination in the later stage. In addition, neuropathy is present in all clinical forms, including those of some contacts.

The diagnosis is essentially clinical, based on a thorough dermatoneurological examination, and in the presence of hypochromic macules, the use of Semmes-Weinstein monofilaments improves its accuracy (32). Current diagnostic tools such as ELISA, PCR, and electroneuromyography (ENMG) have proved to be effective for an early diagnosis of HD and are useful for the evaluation of the efficacy of therapy, but their use is limited in HD, which has been considered a marginalized disease. In addition, these diagnostic methods are only available at referral centers and in teaching and research services (6, 31). Furthermore, pure neural HD, accounting for 5–10% of index cases that present with asymmetric neuropathy in the absence of bacilli in skin smears, remains a diagnostic challenge, often requiring a nerve biopsy, rarely available in the “clusters” and in the areas of higher endemicity (33, 34). Santos et al. (22) demonstrated that, among contacts of patients with HD eligible for a biopsy due to a change in ENMG, 27.8% showed anatomopathological changes suggestive of HD.

In 1977, some authors detected a clearly greater gradual reduction in nerve conduction velocity in the contacts of patients with HD than in HVs, suggesting that a careful (probably improved) method of recording sensory nerve action potentials in the radial cutaneous nerve branch to the index finger could be of help by confirming a diagnosis of leprosy and by detecting the disease in contacts of patients with leprosy before any clinical or bacteriological evidence of leprosy (35).

Neural thickening is one of the three cardinal signs defining a case of HD proposed by the World Health Organization guidelines (36), although only exclusively considering the clinical findings obtained by the palpation technique. When the first signs of neural damage can be noticed, at least 30% of the nerve fibers may show damage (37, 38). HD is a neural disease that may or may not have cutaneous manifestations (2, 3, 39–44). On the contrary, cases of peripheral neuropathy accompanied by neural thickening, with or without cutaneous manifestations, should lead the clinician to suspect the diagnosis of HD (45). Physical examination based on simplified neurological assessment, including palpation of the peripheral nerves, aids in the diagnosis of neural thickening and neuritis; however, this method is subjective, and the earliest cases may not be detected even by well-trained professionals (46, 47).

High-resolution ultrasound (HRUS) can evaluate most peripheral, superficial, and deep nerves and is a validated technique with good reproducibility allowing detailed and accurate examination (48–51). A hand-held ultrasound device can readily identify nerve enlargement in individuals with leprosy, notable in areas with limited healthcare resources because of the portability and low-cost nature of such devices (51, 52).

In the case of pure or primary neural leprosy (PNL), serial scans could be valuable in monitoring treatment and reactions, particularly when it is impossible to determine whether the patient is in remission. In addition, the authors suggest the US as a likely useful tool in the diagnosis of PNH (42, 49, 51).

This study proposes to assess the HRUS of peripheral nerves with an HD protocol as a diagnostic method for asymmetric and fusiform (focal) multiple mononeuropathy by comparing the household contacts of patients with untreated HD with healthy individuals and with those diagnosed with untreated HD.

## Materials and methods

### Ethical statement

This study was approved by the Research Ethics Committee of Hospital das Clínicas, Faculdade de Medicina de Ribeirão Preto, Universidade de São Paulo (protocol number 2.165.032, MH-Brazil and 92228318.1.0000.5440). Written informed consent was obtained from all participants, including the parents and guardians of each participant under 18 years of age. All procedures involving human subjects are in accordance with the ethical standards of the Declaration of Helsinki (1975/2008).

### Sample

Between 2016 and 2020, municipalities from different regions of Brazil (north, northeast, and southeast) whose health professionals were trained by the National Reference Center in Health Dermatology with an emphasis on HD of HCFMRP-USP were selected. In these regions, we randomly selected 176 patients (HD) and 83 household contacts (HHC) from the MB (multibacillary) patients HD by the teams. All of them were Brazilian volunteers to be submitted to peripheral nerve ultrasound. As a comparative sample of healthy Brazilian volunteers, we used 49 healthy individuals (HVs) among the cases of our service, and as a comparative sample of patients diagnosed with HD (HD), we used cases of our service as published by Voltan et al. (53, 54).

Before being submitted to the HD protocol through the HRUS, all HHCs were clinically evaluated by specialist physicians, dermatologists, or leprologists. Exclusion criteria were all individuals with neurological symptoms such as loss of strength, paresthesia, electric shock-like pain, pain or cramps, with a body mass index ≥35.0 kg/m^2^, a diagnosis of metaboli [c]c disease (diabetes mellitus, hypothyroidism or hyperthyroidism) or other peripheral neuropathies, and an amputated limb.

### Clinical evaluation

The clinical evaluation was performed by dermatologists and HD specialists trained by the Ministry of Health program. The teams were not involved in the execution and analysis of the HRUS images.

### Echography of the peripheral nerves

**Figure 1:**
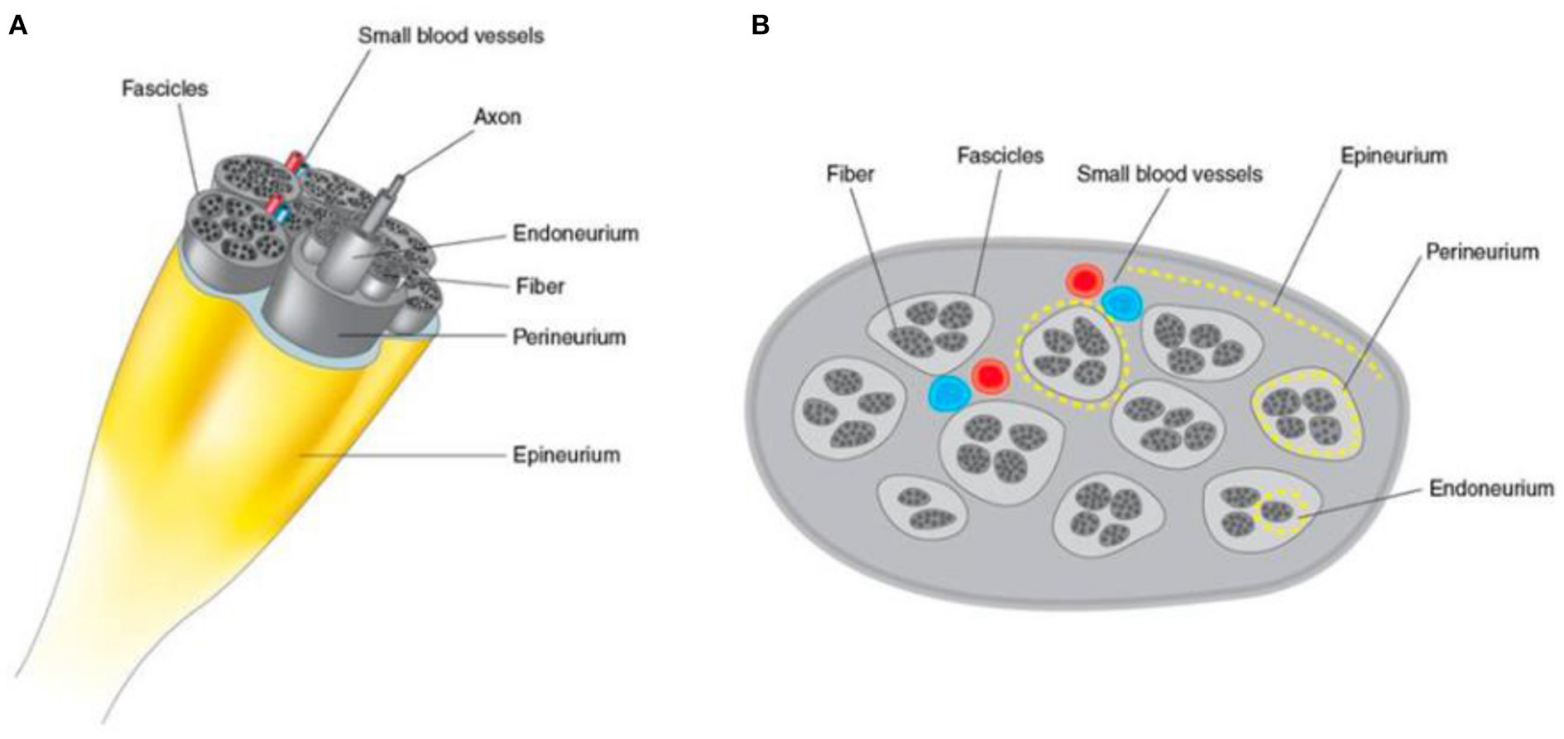
Schematic representation of the cross-sectional view of a normal peripheral nerve and its morphological structures obtained by HRUS. **(A)** 3D view and **(B)** with a honeycomb pattern.

Between 2016 and 2020, a physician from our group with a specialization in US and imaging diagnosis and with extensive experience in the neuromuscular US used portable general US devices with high-frequency linear transducers ranging from 4 to 17 MHz. Each peripheral nerve was scanned in transverse and longitudinal sections, and the cross-sectional area (CSA) of transverse sections was obtained with adjustment of the angle perpendicular to the insonated nerve surface and without pressing the structures. The neural points evaluated were selected due to their proximity to bone anatomical references, facilitating the reproducibility of the method, and because they are known sites for neural compression or more common electrophysiological evaluation, besides being already well-established in the literature. The CSA was measured at these points with a continuous trace, within the hyperechoic borders of the epineurium (Figures 1A, B). For comparison with the literature, all patients underwent echographic evaluation of 10 established neural points, namely, the median nerves in the carpal tunnel (Med CT); the ulnar nerves in the cubital tunnel (UT) and the distal third of the arm (cubital pre-tunnel: 3–5 cm above the medial epicondyle)-(UpT); the common fibular nerves in the topography of the fibular head (CFFH); and the tibial nerves in the tarsal tunnel (T), all bilaterally. Exceptionally, in addition to these, four new points were established for routine focality assessment, i.e., the common fibular nerves proximal to the fibular head (3–8 cm proximal to the head of the fibula)-(CFpFH) and median nerves in the distal third of the forearm (Med pCT) (2–5 cm proximal to the carpal tunnel). The upper limb nerves were evaluated with the patient sitting and with elbows flexed between 60 and 90°. The nerves of the lower limbs were evaluated with the patient sitting or in lateral decubitus with legs slightly flexed between 90 and 130°.

### Statistical analysis

Cross-sectional area values obtained from photographs of the US exams were plotted using the Excel® software and further analyzed using Prism 8 for macOS software. We calculated the means, standard deviations, and medians of absolute CSA values for each of the 14 points of the nerves evaluated (7 on the right side and 7 on the left side). The paired *t*-test was applied to analyze asymmetries. We calculated the indices (Δ) of the differences in absolute CSA values between the right and left sides (ΔCSAs - asymmetry) and of the differences in absolute CSA values between segments of the same nerve (ΔTpT - focality). Neural points were considered to be altered (thickened) when their absolute CSA values were greater than the reference values for normal individuals added to 2 times the standard deviation values (RV + 2 SD). Each neural point was considered to be asymmetric when ΔCSA was greater than its RV + 2 SD, and each neural point was considered to have fusiform or focal thickening when ΔTpT was greater than RV + 2 SD. We also calculated the total number of neural points with increased CSA per individual using the “cont.se” function of the Excel program, as well as the total number of points altered with respect to asymmetry (ΔCSA) and focality (ΔTpT) for each individual. The HV, HHC, and HD groups were compared by the unpaired *T*-test and the non-parametric Mann-Whitney test using the Prism program. To evaluate the discriminatory capacity of the US for the diagnosis of HD neuropathy, the ROC curve was applied to the numbers of altered points according to CSA, ΔCSA, and ΔTpT, which were then compared to the respective numbers obtained for healthy individuals.

## Results

The demographic characteristics, place of birth, and respective anti-PGL1 serology results of the population sample evaluated are described in Table 1.

**Table 1:** Distribution of the population sample of HHDs (contacts of patients with HD) by sex, age group, region of origin, and anti-PGL-I data.

**Variables**		***N***	**%**
Sex	Male	34	41.46
	Female	49	58.54
Age (years)	04 a 15	14	17.07
	15 a 60	69	82.93
Region of origin	North	48	58.54
	Northeast	11	13.41
	Southeast	23	28.05
Anti-PGL 1	Negative (0.47 ± 0.32)	20	24.39
	Positive (2.85 ± 3.96)	8	9.76
	Not rated	54	65.85

For comparison of the values obtained for the patients, we considered the values established by Voltan et al. (54) as normality standards (healthy individuals—none of whom had any known contact with leprosy) for CSAs in the Brazilian population.

We have been following 11 HHCs from the southwest—Jardinópolis/SP for 3 years, 5 of whom have developed HD with neuropathy.

### Absolute CSA values (mm^2^) of the peripheral nerves and their indices

The 83 HHCs were evaluated by bilateral HRUS of the following peripheral nerves: Med CT, UT UpT, CFFH, and T. Based on initial observations during fieldwork, additional evaluation of new analysis points of the Med Ab and CFpFH was established as routine in the examinations. Thus, the sample number (n) varied, with measurements of 24 (29.2 %) HHCs being obtained for Med pCT and 38 (46.3 %) HHCs for CFpFH. In 21 HHCs, 14 neural points (MCT, Med pCT, UT, UpT, CFFH, CFpFH, T) were evaluated.

The means, standard deviations, and median absolute CSA values in mm^2^ and the values of the differences between the right and left sides (ΔCSAs/ΔAsymmetry) and between two points of the same nerve (Δ TpT/Δfocality) are listed in Table 2 according to age group.

**Table 2:** Distribution of ultrasound measurements (CSA, ΔCSA, ΔTpT) according to age range in HHCs and upper normal limit in HVs.

**Variables**	**Age range (years)**	**04–15**	**15–30**	**31–45**	**46–60**		**Upper normal Limit (HVs)**
						**(15–60 y)**	**(mean** + **2 SD)**
	(*n*) Men	7	14	10	3	27	–
	(*n*) Women	7	15	19	8	42	–
	(*n*) Total (right + left)	28	58	58	22	138	–
	Mean ± SP [median]	10.3 ± 2.9 [11]	23.2 ± 4.7 [22]	36.3 ± 4.0 [37]	52.3 ± 4.4 [52]	29.2 ± 13.5 [29]	–
	**Site**	**Mean** ±**SD [median]**					
Peripheral nerve CSA (mm^2^)	Med CT	7.2 ± 2.0 [7.1]	10.3 ± 10.3 [9.0]	10.7 ± 3.0 [10.0]	12.9 ± 4.0 [11.7]	10.9 ± 3.3 [10.0]	10.2
	UT	5.2 ± 1.4 [5]	7.0 ± 1.8 [7]	8.0 ± 1.9 [7.9]	9.1 ± 4.5 [8]	7.7 ± 2.3 [7.5]	9.8
	UPT	5.3 ± 1.5 [5.2]	6.5 ± 1.5 [6.6]	7.2 ± 1.7 [7]	8.2 ± 3.2 [7.0]	7.0 ± 2.0 [7]	9.3
	CFFH	9.4 ± 2.7 [10]	15.3 ± 4.2 [14.6]	17.5 ± 5.1 [16.5]	15.4 ± 4.1 [14.4]	16.2 ± 4.6 [15.3]	18.3
	T	6.4 ± 1.8 [6.9]	8.1 ± 2.4 [8]	9.8 ± 2.8 [9.8]	9.3 ± 3.4 [8.0]	9.0 ± 2.8 [8.7]	–
ΔCSA (mm^2^)	Med CT	1.3 ±1.3 [1.0]	2.0 ± 1.2 [2.0]	1.4 ± 1.1 [1]	2.8 ± 3.5 [1.5]	1.8 ± 1.8 [1.2]	2.2
	UT	0.7 ± 0.6 [0.9]	1.3 ± 1.5 [1]	1.3 ± 1.1 [1.0]	1.8 ± 1.2 [2.0]	1.4 ± 1.2 [1.0]	3.1
	UPT	0.7 ± 0.9 [0.5]	1.3 ± 1.2 [1]	1.5 ± 1.3 [1.0]	1.9 ± 1.8 [1]	1.5 ± 1.4 [1.0]	1.4
	CFFH	1.7 ± 1.7 [1]	2.9± 3.2 [1.7]	3.2 ± 3.1 [2]	1.9 ± 1.4 [2]	2.8 ± 2.9 [2]	2.3
	T	1.0 ± 1.0 [1]	1.7 ± 1.8 [1.4]	1.8 ± 2.0 [1.0]	1.4 ± 1.5 [1.0]	1.7 ± 1.8 [1.0]	–
ΔTpT (mm^2^)	Ulnar (UT and UPT)	0.7 ± 0.6 [0.9]	1.1 ± 0.9 [1]	1.3 ± 1.4 [1]	2.1 ± 1.9 [1.5]	1.3 ± 1.3 [1]	2.6
**Analysis of new neural sites**							
	Total *n* = 48	6	10	26	6	42	–
Peripheral nerve CSA (mm^2^)	Med pCT	4.9 ± 0.87 [4.6]	7.9 ± 2.3 [7.2]	8.4 ± 2.0 [8.2]	11.6 ± 3.6 [10.3]	8.7 ± 2.6 [8.3]	–
ΔCSA (mm2)	Med pCT	0.7 ± 0.7 [0.4]	1.5 ± 1.1 [0.9]	1.3 ± 1.7 [0.7]	2.8 ± 3.3 [1.7]	1.5 ± 1.8 [0.9]	–
ΔTpT (mm^2^) -	Median (CT and pCT)	0.9 ± 0.9 [0.8]	1.6 ± 1.0 [1.4]	2.8 ± 2.2 [2.8]	4.0 ± 2.9 [3.8]	2.7 ± 2.2 [2.3]	–
	Total *n* = 76	10	28	32	6	66	–
Peripheral nerve CSA (mm^2^)	CFpFH	7.4 ± 2.2 [7.0]	13.9 ± 3.4 [14.8]	17.0 ± 4.4 [15.8]	19.7 ± 5.7 [18.3]	15.9 ± 4.4 [15.5]	–
ΔCSA (mm2)	CFpFH	1.1 ± 0.9 [1.0]	2.4 ± 2.2 [1.3]	2.8 ± 2.3 [2.5]	6.3 ± 3.6 [6.3]	2.9 ± 2.6 [2.0]	–
ΔTpT (mm^2^)	Fibular common (FH e pFH)	0.6 ± 0.7 [0.3]	2.9 ± 2.8 [2]	2.8 ± 2.8 [2.0]	3.9 ± 2.6 [4.3]	2.9 ± 2.8 [2.0]	–

Data are reported as mean ± standard deviation and median (mm^2^). (*n*), number; Med, median; CT, carpal tunnel; pCT, proximal to the carpal tunnel; UT, ulnar tunnel; UPT, ulnar pre-tunnel; CFFH, common fibular in the fibula head; T, tibial; CFpFH, common fibular proximal to the fibular head; ΔCSA, difference between the right and left nerves at the same site of assessment; ΔTpT, difference between the same nerve at different sites; HVs, healthy volunteers; HHCs, household contacts of patients with HD.

### Comparative analysis of healthy individuals, household contacts of patients diagnosed with HD, and patients with HD

We compared 49 healthy individuals (98 neural points) with 69 household contacts (HHCs) of patients diagnosed with HD (138 neural points) and with 176 patients diagnosed with HD (352 neural points) in the age range of 15–60 years.

Since the samples were unpaired and did not have parametric distribution, both the absolute values of the peripheral nerve CSAs and the values of the differences between the CSAs were compared by the non-parametric Mann-Whitney test.

**Figure 2:**
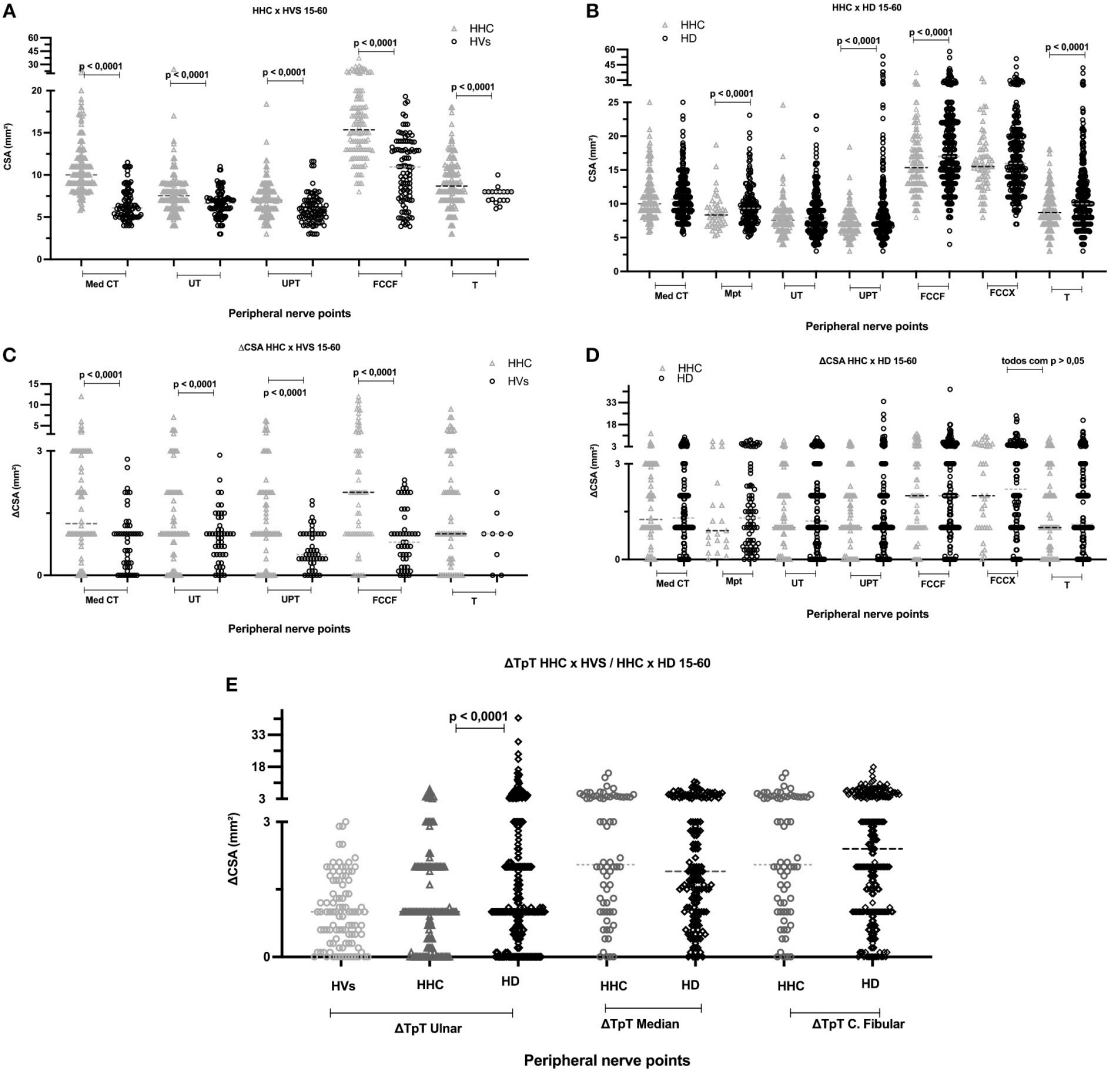
Absolute CSA values (mm^2^) of peripheral nerve sites for HVs and HHC [**(A)** up on the left]; absolute CSA values of peripheral nerves sites for HHC and HD [**(B)** up on the right]. ΔCSA (mm^2^) of right and left sides at the same assessment site for HVs and HHC [**(C)** middle on the left]; ΔCSA (mm^2^) of right and left sides at the same assessment site for HHC and HD [**(D)** middle on the right]. ΔTpT (mm^2^) of the different sites of the same nerve for HVs, HHC, and HD [**(E)** below]. (n), number; Med, median; CT, carpal tunnel; UT, ulnar tunnel; UPT, ulnar pre-tunnel; CFFH, common fibular nerve in the fibula head; T, tibial; MpT, median nerve in the proximal carpal tunnel; CFpFH, common fibular nerve proximal to the fibular head; CSA, cross-sectional area; ΔCSA, difference between the right and left nerves at the same site of assessment; ΔTpT, difference between the same nerve at different sites; HVs, healthy volunteers; HHC, household contacts of patients with HD; HD, Hansen's disease.

Our results showed higher medians and means of the absolute values of the CSAs of peripheral nerves and greater asymmetries (ΔCSA) in the HHCs compared to the healthy volunteers at all points amenable to comparison, except for the ΔCSA of the tibial nerve, which was equal for the two groups. There was no difference in focus (ΔTpT) of the ulnar nerve between these groups although the mean ± standard deviation showed higher values for the household contacts (HHC = 1.3 ± 1.3 x HVs = 1.0 ± 0.8). The median and mean absolute values of peripheral nerve CSAs were higher in HDs than in HCCs at almost all points except for the Med Ct and UT neural points, which were equal. Asymmetry (ΔCSA) did not differ between groups at any point. The mean ± SD focality (ΔTpT) values of HCC and patients with HD were 2.7 ± 2.2/2.6 ± 2.2 for the median nerve, 2.9 ± 2.7/3.3 ± 2.9 for the common fibular nerve (*p* > 0.05), and 1.3 ± 1.3/2.2 ± 3.9 for the ulnar nerve (*p* < 0.0001), respectively, as demonstrated in Figure 2.

**Figure 3:**
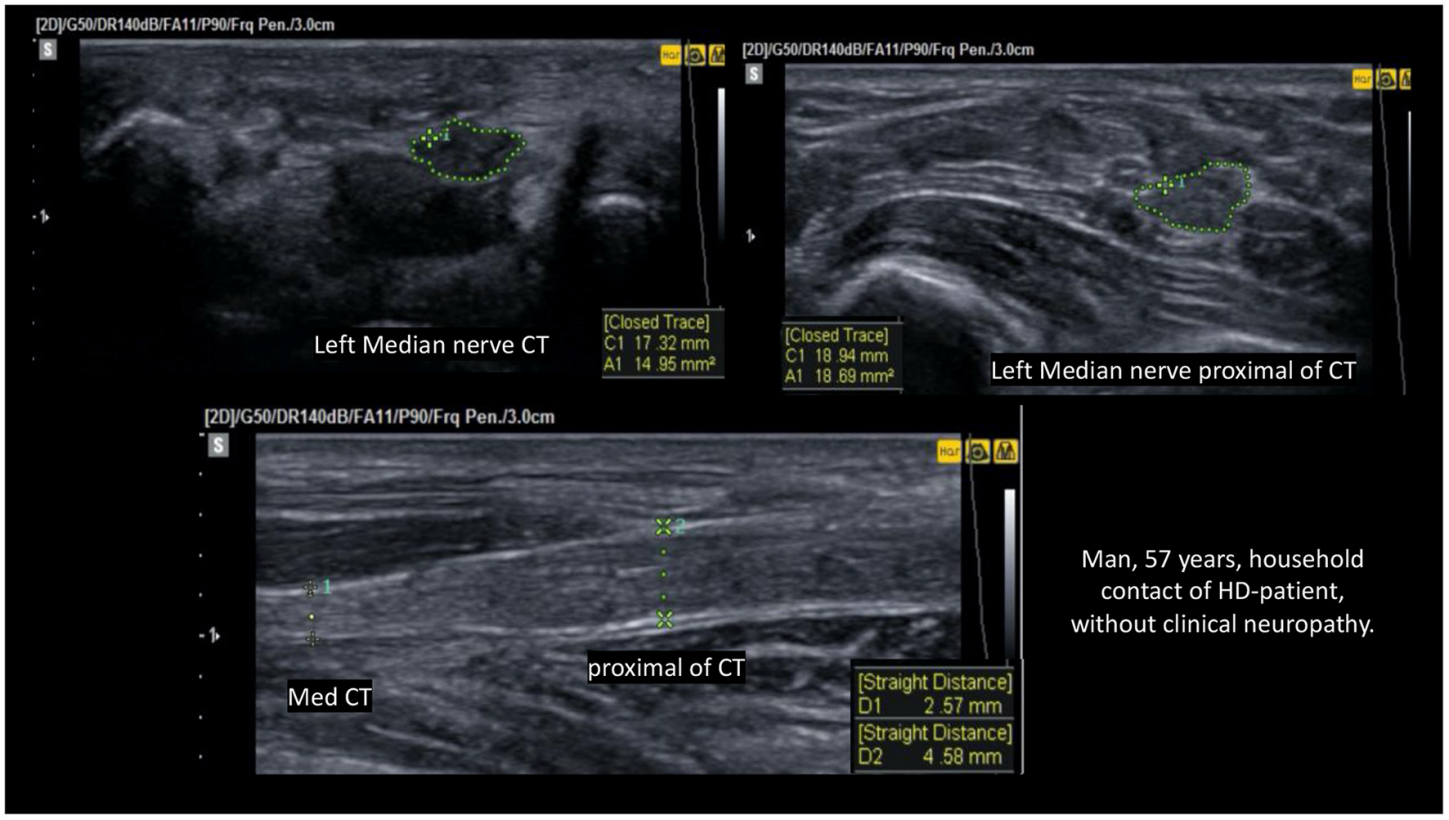
Fusiform thickening of the left median nerve regarding focality (ΔTpT) CSA pCT > CSA CT; ΔTpT = 18.69 −14.95 = 3.7 mm^2^ > ULV (upper limit value).

In 29 HHCs and 91 patients with HD, we were able to discriminate the ΔTpT (focality) of the median nerve between thickening in the carpal tunnel or proximal to the carpal tunnel, and we obtained greater nerve thickening proximal to the carpal pre-tunnel in 2 HHCs (7%) x 20 HD (22%) (Figure 3) and greater nerve enlargement on the carpal tunnel in 19 HHCs (65%) x 49 HD (53%). The chi-square value was 8.9, with *p* < 0.05.

### Absolute number and percentage of thickened nerves per individual

The nerves were considered to be enlarged when their CSA was greater than the mean ± 2 SD of the normal values established in the study by Voltan et al. (54).

To determine a possible relationship between the categorical variables, thickened nerves, neural points with asymmetry between right and left sides (ΔCSA), and nerves with the difference between segments of the same nerve (focality/ ΔTpT), we compared by the chi-square test and ROC curve household contacts of untreated patients diagnosed with HD, healthy individuals, and patients diagnosed with HD.

When we compared the mean, standard deviation, and median [Md ± SD (M)] values of the index of the number of nerves with increased CSA per individual, we detected a significant difference (*p* < 0.05) between all groups: healthy subjects [0.04 ± 0.09 (0)], HHC [0.27 ± 0.26 (0.2)], and HD [0.41 ± 0.29 (0.4)]. Regarding ΔCSA altered beyond normality, we detected the following differences: HVs [0.02 ± 0.08 (0)] x HHCs [0.3 ± 0.23 (0.2)] and HVs x HD [0.32 ± 0.24 (0.4)]. Also, we detected higher than normal neural focality (ΔTpT) in the HD group [0.24 ± 0.32 (0)] compared to HHCs [0.13 ± 0.27 (0)] and in the HHC group compared to HVs [0.03 ± 0.12 (0)].

### HHCs vs. HVs

**Figure 4:**
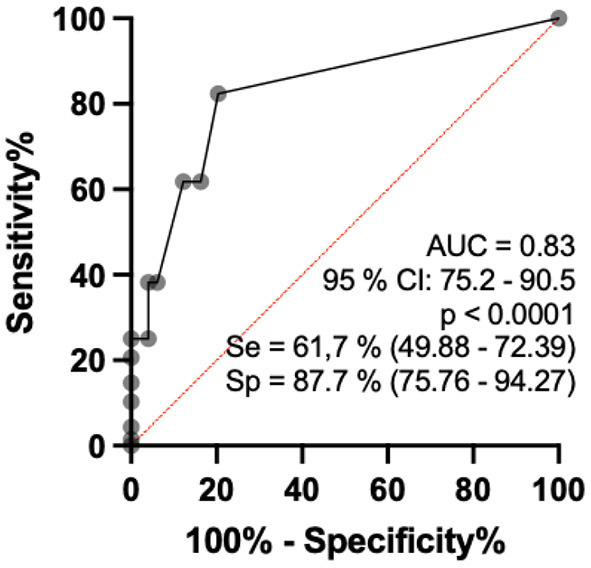
Analysis by the ROC curve of the percentage indices of altered nerves among the nerves evaluated per individual comparing the groups of healthy individuals and contacts of patients with HD aged 15–60 years.

When comparing healthy individuals to contacts of patients diagnosed with HD aged 15–60 years, we observed that the percentage of thickened nerves per nerve evaluated per individual was higher in contacts of patients with HD than in healthy subjects (*p* < 0.0001). Analysis by the ROC curve revealed that the AUC was 83.0 (95% CI: 75.4–90.5%, *p* < 0.0001), and when the percentage of altered nerves among those evaluated was higher than 16.5%, sensitivity reached 61.7% (CI: 49.9 to 72.4) and specificity reached 87.8% (75.8 to 94.3), with a relative risk of 5.0 (Figure 4).

**Figure 5:**
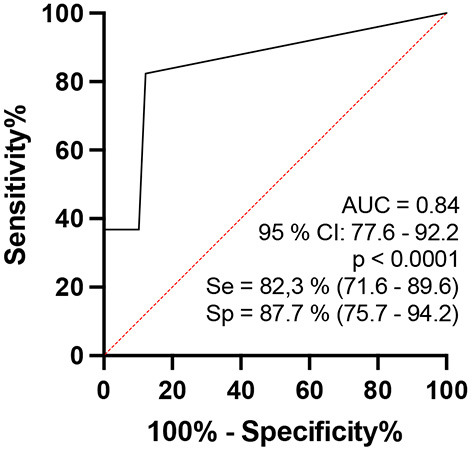
ROC curve analysis of the percentage indices of the number of asymmetries (ΔCSA) of the evaluated neural points considered to be altered per individual when comparing the groups of healthy persons and contacts of patients with HD (b – on the right).

Regarding the number of asymmetries between neural points (ΔCSA) per individual defined as altered (greater than the reference mean + 2DP) by the ROC curve, comparison between healthy individuals and contacts of patients diagnosed with leprosy aged 15–60 years revealed a ROC curve of 84.9 (95% CI: 0.77–0.92, *p* < 0.0001) and when the percentage index of neural points defined as asymmetric was >10% of the points evaluated, sensitivity was 82.3% (71.6–89.6) and specificity was 87.8% (75.76–94.27), with a relative risk of 6.72, as shown in Figure 5.

**Figure 6:**
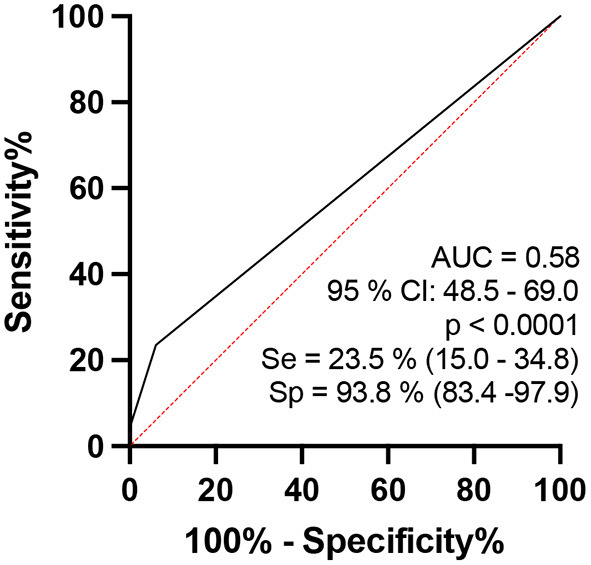
ROC curve analysis of the percentage indices of the number of focalities (ΔTpT) of the evaluated neural points considered to be altered per individual when comparing the groups of healthy persons and contacts of HD patients aged 15–60 years.

In the analysis regarding the number of focalities among the points of the same nerve (ΔTpT) defined as altered (greater than the reference mean ± 2DP) per individual by the ROC curve, when comparing healthy individuals to contacts of patients diagnosed with HD aged 15–60 years, the area under the curve was 58.2 (95% CI: 0.50–0.65, *p* < 0.05), and when the percentage index of neural points defined as having focality was >25% of the points evaluated, sensitivity was 39.7% (32.8–47.1) and specificity was 76.4% (65.1–84.9), with a relative risk of 1.69, as shown in Figure 6.

### HHCs x HDs

**Figure 7:**
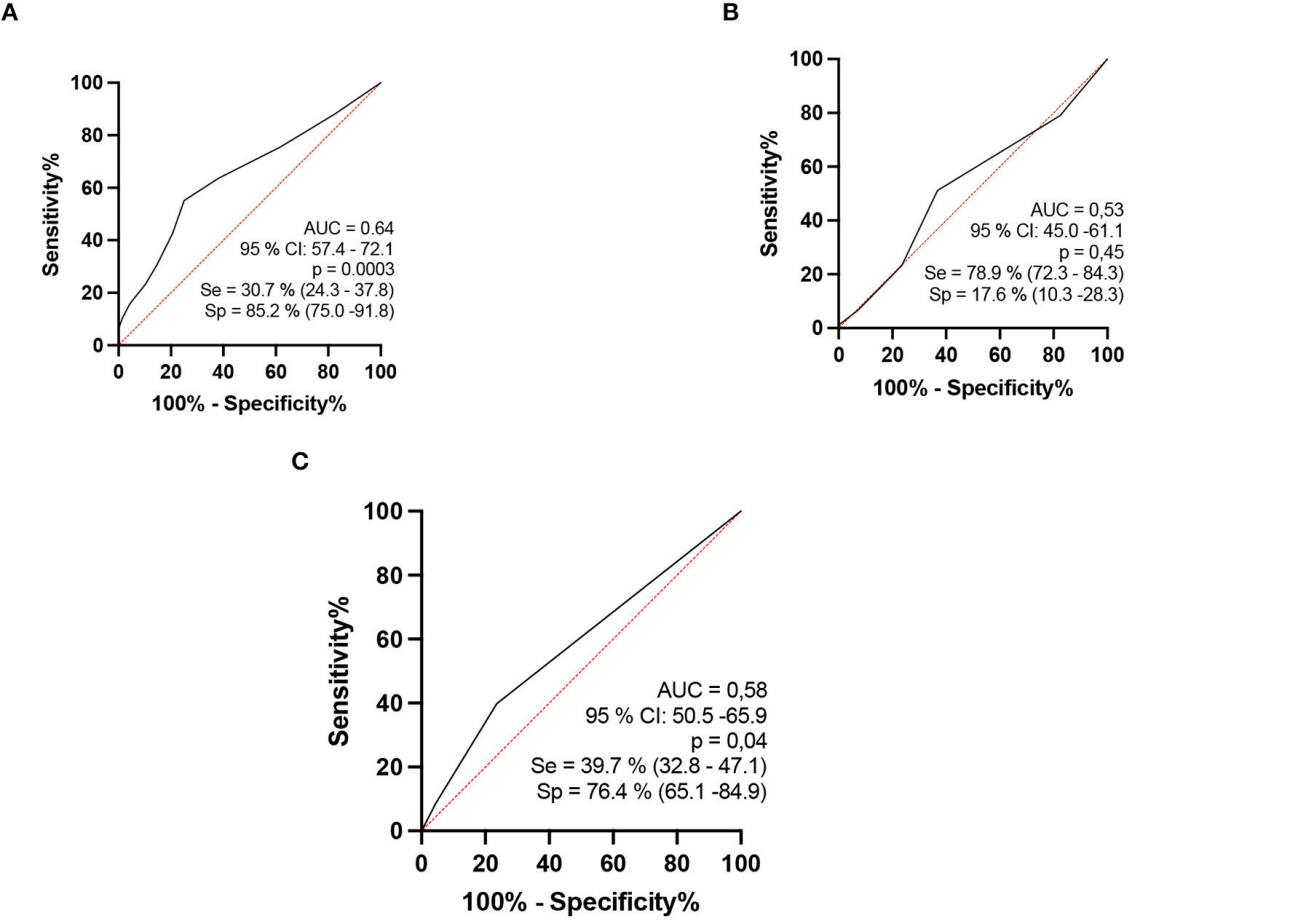
Analysis by the ROC curve comparing HHC and patients with HD aged 15–60 years: percentage indices of altered nerves among the nerves evaluated per individual [**(A)** up on the right]. The percentage indices of the number of asymmetries (ΔCSA) [**(B)** up on the left]. The percentage indices of the number of focalities (ΔTpT) [**(C)** as mentioned later].

When we compared contacts of patients diagnosed with HD to patients aged 15–60 years, we observed that the percentage of thickened nerves per nerve evaluated per individual was higher among patients with HD than among their contacts (*p* = 0.0003). Considering analysis by the ROC curve, we observed that the AUC was 64.8 (95% CI: 0.57–0.72, *p* = 0.0003) and when the values of the percentage of altered nerves among those evaluated were higher than 55%, sensitivity reached 30.6% (CI: 24.3–37.8) and specificity reached 85.3% (75–91.8), with a relative risk of 2.0 (Figure 6). Considering the percentage index of specificity found (82.3%), for a binomial analysis, we divided our samples into individuals with up to 6 nerves altered (<6) and individuals with more than 6 nerves altered (≥6). The chi-square value with Yates' correction was 6.5 (*p* < 0.05), and we found that individuals with six or more thickened nerves had a relative risk of 0.48 and an odds ratio of 0.39 (Figure 7).

## Discussion

We found only one article (55) in the literature that evaluated by the US a peripheral nerve of contacts of the patient diagnosed with HD (PubMed; keywords: leprosy + contacts + ultrasound + nerves), and three other authors suggested that the US can be used for the evaluation of HD contacts (55–57). To the best of our knowledge, our sample of the number of nerves evaluated with HRUS of peripheral nerves in HHC and patients must be the largest in the world.

When we analyze healthy individuals, contacts of patients diagnosed with HD, and patients with HD, we note neural involvement even before the individuals develop the disease. There is thickening with increased CSA in all nerves of HD contacts compared to healthy individuals although this thickening is lower compared to patients with HD. Our results support the findings of other studies by objectively demonstrating that the contact factor increases the risk of developing HD (6, 11, 13, 19, 22–24); furthermore, peripheral nerve US with an HD protocol demonstrated that the neuropathy secondary to HD is a multiple peripheral mononeuropathy, confirming the results of other authors (22, 40, 43, 44, 58–64).

Moreover, given the natural history of the disease, these data show the primary neural involvement of HD detected with objective diagnostic imaging criteria. We question whether this early peripheral nerve involvement might be a subclinical phase of the disease or a host immune response. In any case, if there is nerve involvement, it is to be expected that there was an interaction between the pathogen and host, although not all individuals do develop the disease (9–13, 16).

**Figure 8:**
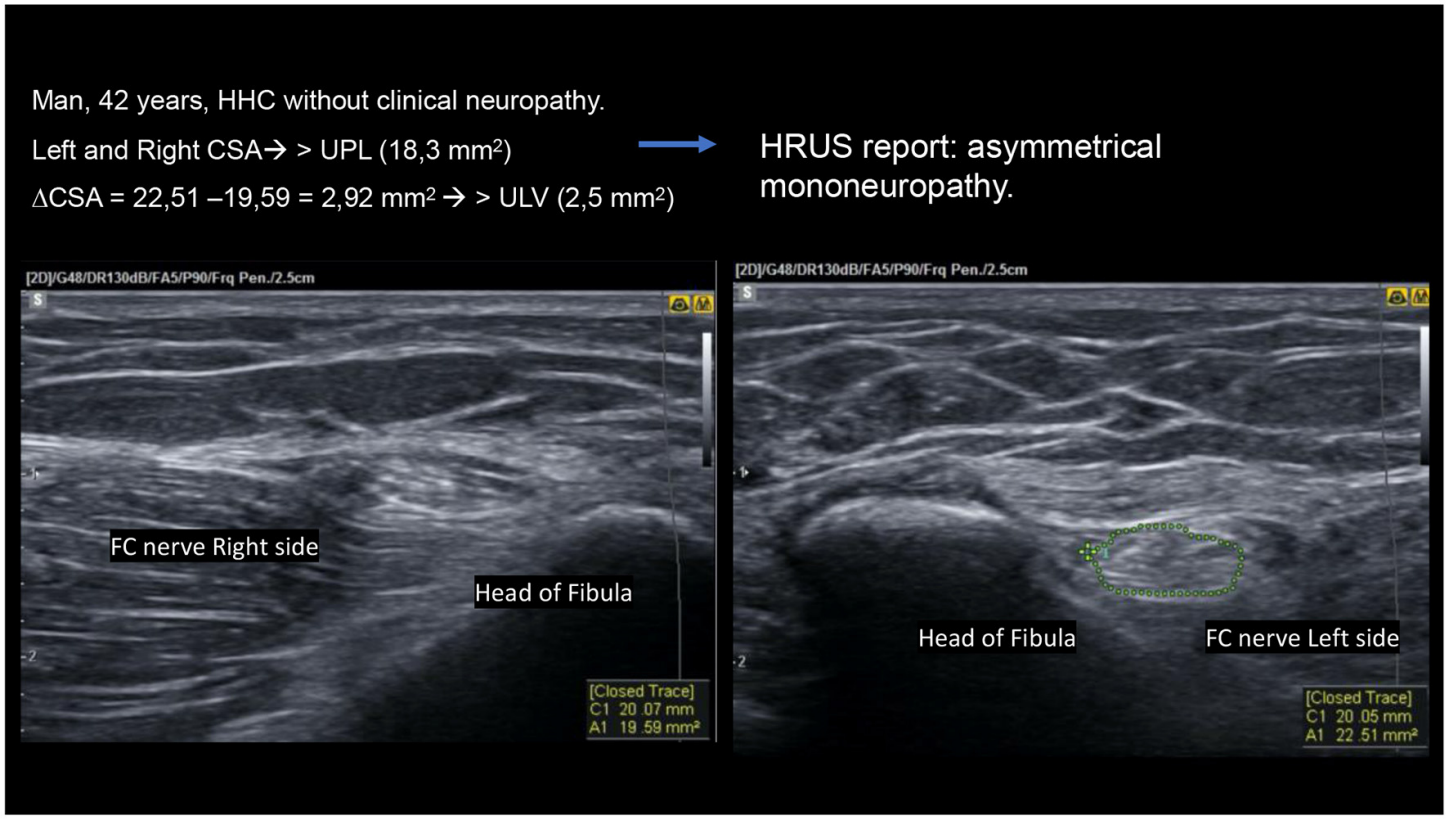
High-resolution ultrasound (HRUS) images of the same HHC of a patient with HD with asymmetrical mononeuropathy of the common fibular nerve on the head of the fibula.

Regarding the morphological aspects of the behavior of the disease with respect to the involvement of peripheral nerves, when we analyze the asymmetry criteria, we can observe that the contacts of MB-patients with HD already have more asymmetrical nerves (median, ulnar and fibular), with above normal differences between the right and left sides when compared to healthy individuals, as observed by Frade et al. (40) and Lugão et al. (43) and also when healthy individuals are compared to patients. However, since asymmetry (ΔCSA) was already installed in household contacts, this criterion showed no difference between HHC and patients (Figure 8).

Regarding focality within the same nerve, there was no difference between healthy subjects and contacts in the ulnar nerve; however, there was a difference between contacts and patients, demonstrating the late occurrence of this event in the natural history of the disease, as observed by Bathala et al. (59, 65) and Frade et al. (40) when comparing healthy individuals to those diagnosed with HD. The ΔTpT focality of the median nerve and the common fibular nerve was similar in HHCs and patients with HD.

**Figure 9:**
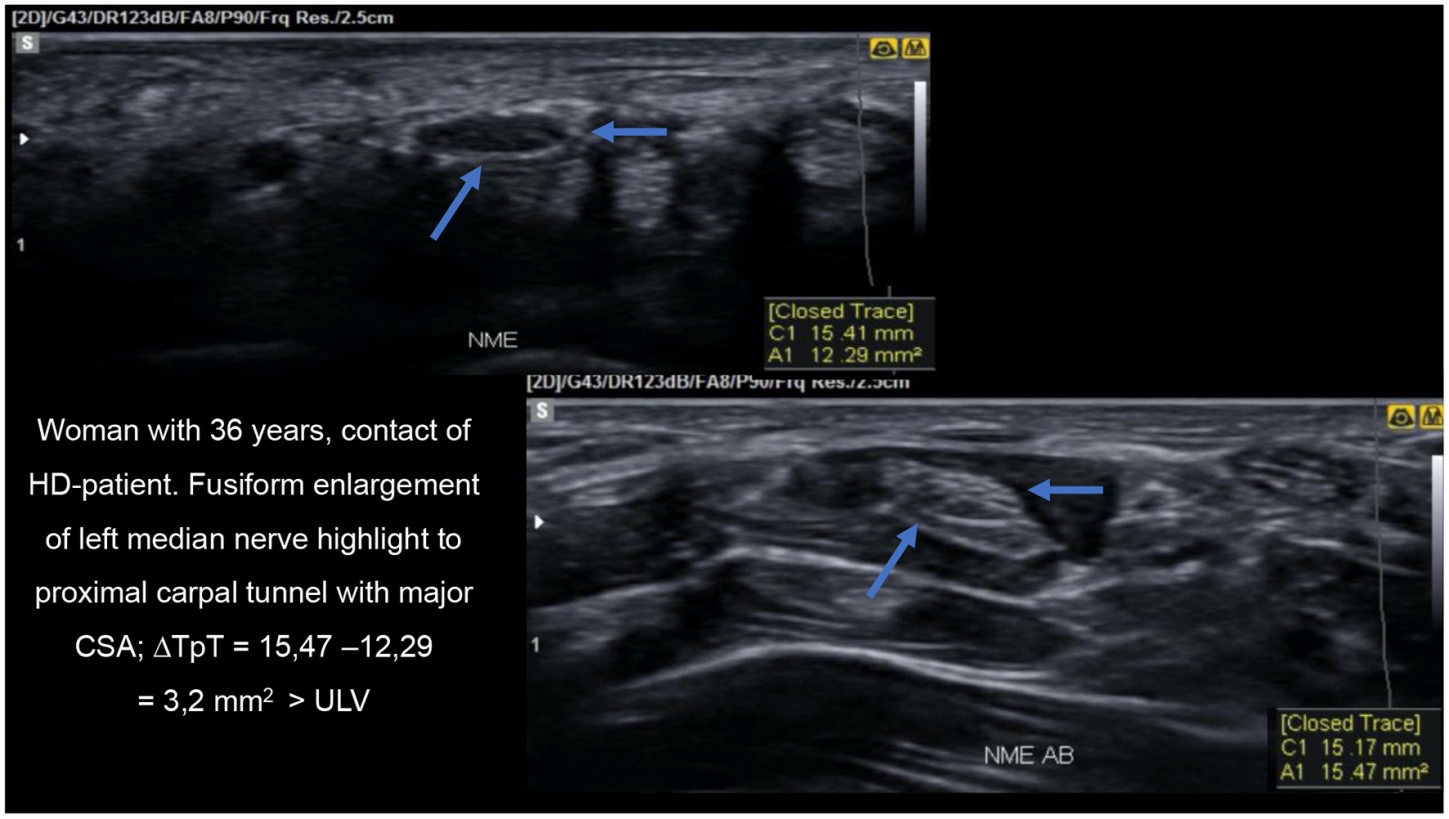
HRUS image demonstrating thickening of the median nerve proximal to the carpal tunnel on transverse view.

Nagappa et al. (66) concluded that a median nerve enlargement of 2.0 cm proximal to the distal wrist crease distinguishes leprosy from carpal tunnel syndrome (CTS). In our study, we showed that patients with HD and their contacts had equal ΔTpT values in the median nerve, and the proportion of HHC with median nerve thickening in the carpal pre-tunnel had 4.1 OR with 90% specificity and positive predictive value compared to patients with HD. Thus, given the difference between healthy and sick subjects, we could characterize focality as a more specific marker of the disease (Figure 9). We would consider the asymmetry criteria to be more sensitive and probably of earlier detection for the characterization of peripheral nerve involvement in the evolutive phases of HD. This finding also shows that serial evaluation of leprosy contacts is a unique tool for assistance programs in public administration, being of help for an early diagnosis and thus contributing to the elimination of the disease (67).

A meta-analysis of the accuracy of diagnostic tests for HD (68) that included 78 studies concluded that, although test accuracy appears reasonable, the studies suffered from heterogeneity and poor methodological quality, most of them evaluating the detection of IgM antibodies against phenolic glycolipid-I by ELISA with 63.8% sensitivity (95% CI: 55.0–71.8) and 91.0% specificity (95% CI: 86.9–93.9) in 39 of the studies while the sensitivity of qPCR (five studies) was 78.5% (95% CI: 61.9–89.2) and specificity was 89.3% (95% CI 61.4–97.8). The sensitivity of conventional PCR (17 studies) was 75.3% (95% CI 67.9–81.5) and specificity was 94.5% (95% CI: 91.4–96.5). Regarding peripheral nerve ultrasonography in the evaluation of HD neuropathy, several studies have considered it as a method with good efficacy, reproducibility, and diagnostic accuracy, besides its accessible cost and availability, even for point of care (40, 43, 47, 49, 59, 69–76).

Healthy individuals had nerves without thickening compared to contacts who had up to 20% of nerves with thickening and to patients who had up to 40% of nerves with increased CSA, results similar to those reported by Wilder-Smith et al. regarding neural dysfunction of peripheral nerves documented by ENMG, which was greater in patients with HD, followed by HHC and HVs (77). The same applies to the number of asymmetries per individual which was higher in HD compared to contacts and healthy individuals.

We also evaluated 4 new neural points in 21 contacts and 78 patients, for a total of 14 rather than 10 points. The AUC of the sum of the thickened nerves increased, thus also increasing the sensitivity and specificity of peripheral nerve US with a protocol for HD evaluation. However, increasing the number of neural points in the evaluation no longer revealed differences in asymmetry and focality between HHC and patients with HD, with the two groups being equal.

About the limitations of our study, perhaps a prospective study with an evaluation of the peripheral nerves of the contacts that show the neural thickening documented by the peripheral nerve US examination may bring more information regarding the development of the pathology. Another limitation of our study was that we did not have data on the ΔTpT (Δfocality) of the median and common fibular nerves of HVs. The data presented could also reflect on the individual's immunity acting against the nerve and promoting thickening and asymmetry, or the subclinical infection itself.

## Conclusion

Peripheral nerve US with a protocol for HD (median, ulnar, common fibular, and tibial nerves) revealed HD as an asymmetric and/or focal peripheral multiple mononeuropathy.

Considering HRUS findings, if asymmetric multiple mononeuropathy signs (thickening or asymmetry) are detected in at least 20% of the nerves evaluated in the HHCs, these findings could already indicate evidence of HD neuropathy. Thus, if more nerve points are assessed in HHCs (14 instead of 10), these individuals become more like patients with HD according to the nerve thickening determined by HRUS, which should be a cutting-edge tool for an early diagnosis of leprosy cases.

Neural hypertrophy detected by HRUS in all peripheral nerve points seems to differentiate contacts from healthy volunteers, but the enlargement of three of seven neural points (median CT, ulnar-tunnel, and commun fibular nerve in pre-head fibula) was similar to that of patients with HD. Asymmetry of peripheral nerves was greater in the HHC group at all neural points compared to healthy volunteers, except in the tibial point, and was similar when compared to patients with HD at all points evaluated. Both findings could constitute important alerts for early and/or subclinical HD, mainly asymmetry, with 82.3% sensitivity and 87.7% specificity, respectively.

Finally, the peripheral nerve focalities of the median and common fibular nerves were similar for HHC and patients with HD, except for the ulnar nerve whose values were higher in patients with HD than in HHC. These data could also suggest important findings for the median and common fibular nerves of individuals with early and/or subclinical HD, which can be properly assessed only by HRUS, while ulnar nerve focality seems to be a late event in the natural course of the HD.

## Data availability statement

The raw data supporting the conclusions of this article will be made available by the authors, without undue reservation.

## Ethics statement

The studies involving human participants were reviewed and approved by Ethics Committee of Hospital das Clínicas da Faculdade de Medicina de Ribeirão Preto da Universidade de São Paulo. Written informed consent to participate in this study was provided by the participants' legal guardian/next of kin.

## Author contributions

GV and MF contributed on all stage of this study, conception and design of the study, performed the statistical analysis, manuscript revision, read, and approved the submitted version. FB, ND, CMLS, ML, JS, JB, CGS, GC, and MD contributed to conception and design of the study, to data collection, obtaining, analyzing and interpreting data, and approved the submitted version. All authors contributed to the article and approved the submitted version.
