# Supplementary material for: Silent peripheral neuropathy determined by high-resolution ultrasound among contacts of patients with Hansen's disease
Source: Front Med (Lausanne). 2023 Jan 17;9:1059448. doi: 10.3389/fmed.2022.1059448 (PMC9887334; doi:10.3389/fmed.2022.1059448)

Median Nerve
Fibular Commun nerve
HHCs - Frontiers

Silent peripheral neuropathy among contacts of Hansen’s disease patients by high-resolution ultrasound.

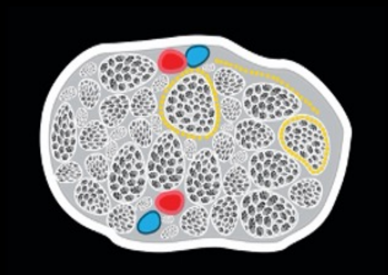

Pattern A or healthy “honey-comb”.

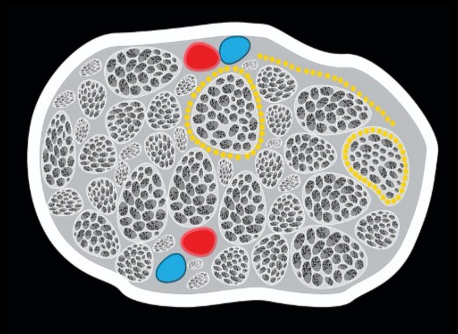

Pattern B or thickened

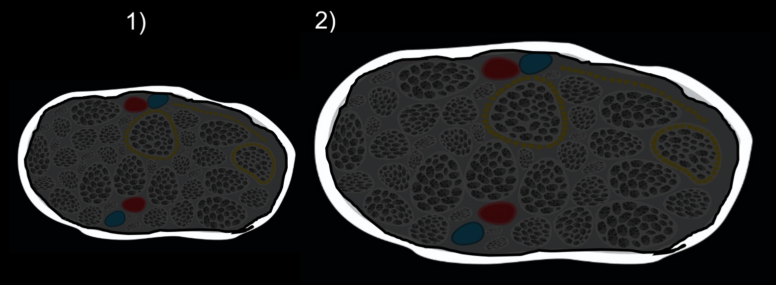

Pattern C:
1) hypoechoic without enlargement ;
2) hypoechoic with enlargement

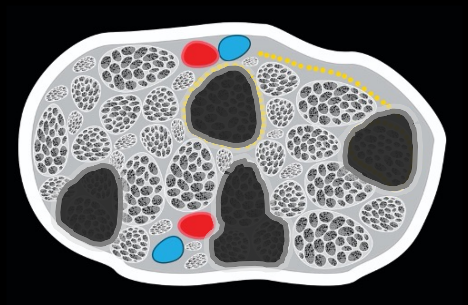

Pattern D ou Scolard modified by Voltan,G and Frade, MCF: thickened with hypoechoic focal distention of fascicles.

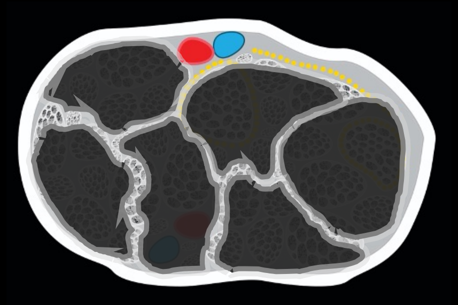

Pattern E: thickened with diffuse hypoechoic distention of fascicles.

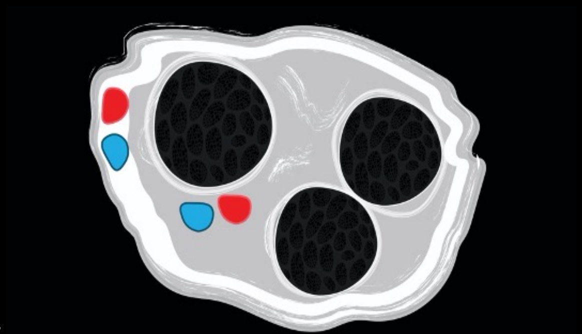

Pattern F: thickened, heterogeneous with thickening of the perineurium and epineurium and distention of fascicles

ORIGINAL RESEARCH article
Front. Med., 09 September 2022
Sec. Dermatology
https://doi.org/10.3389/fmed.2022.985252

Man, 42 years, HHC without clinical neuropathy.

Left and Right CSA→ > UPL (18,3 mm^2)

ΔCSA = 22,51 –19,59 = 2,92 mm^2 → > ULV (2,5 mm^2)

HRUS report: asymmetrical mononeuropathy.

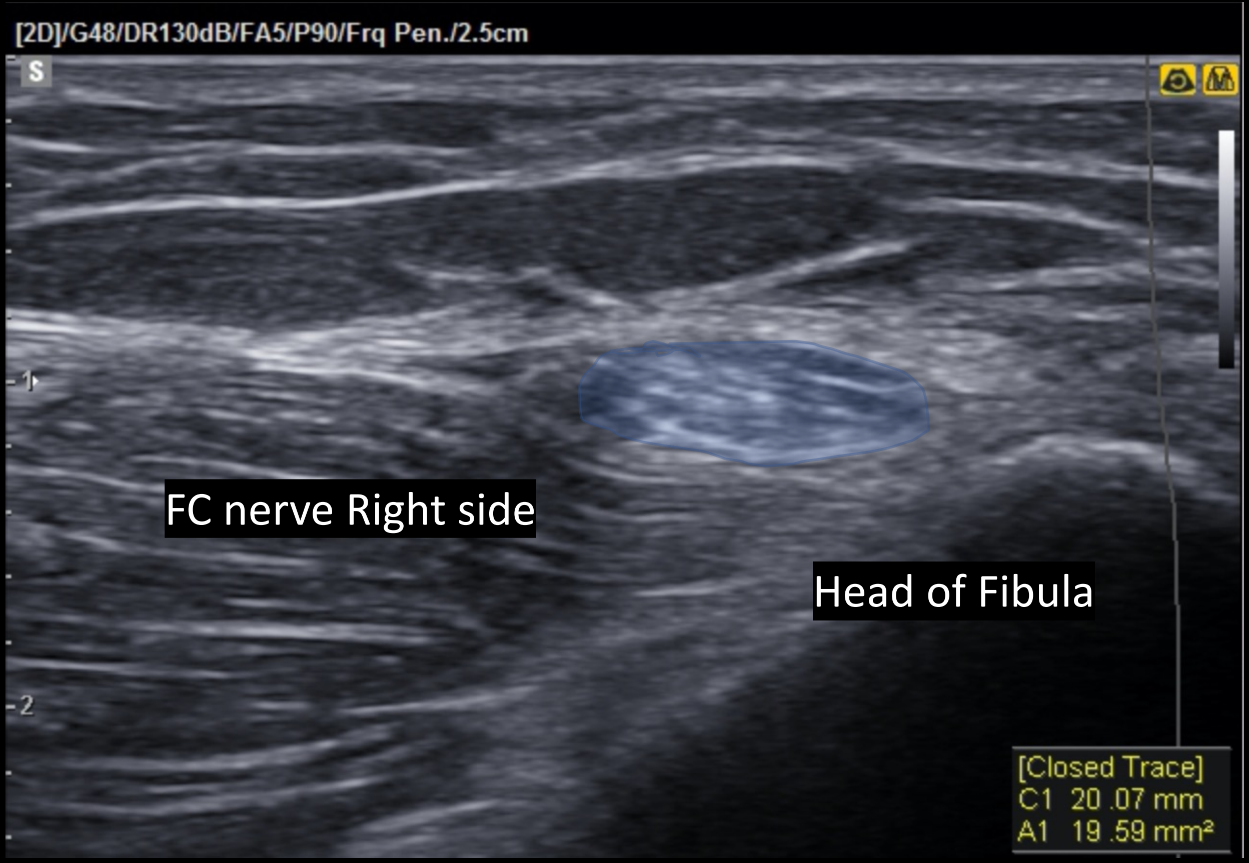

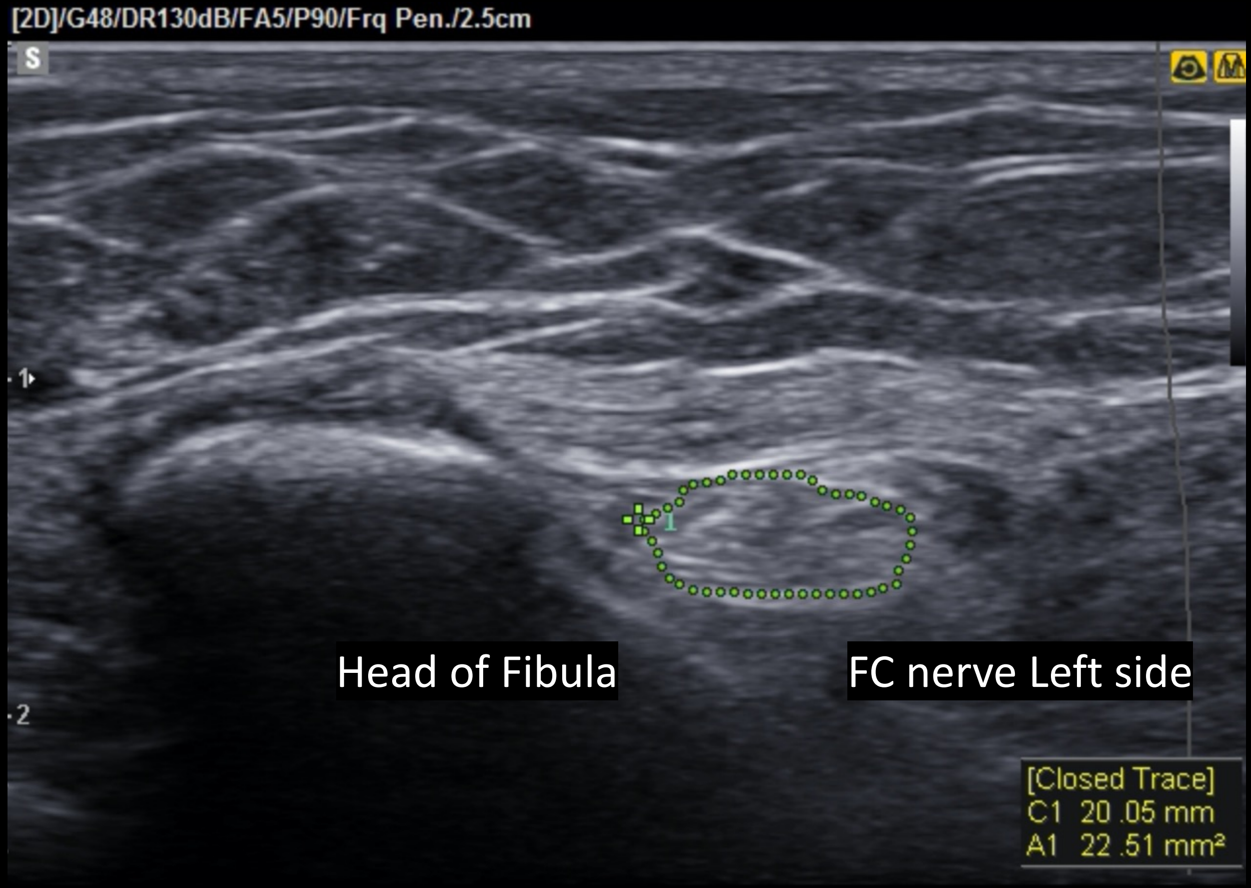

Right - Fibular commun Nerve

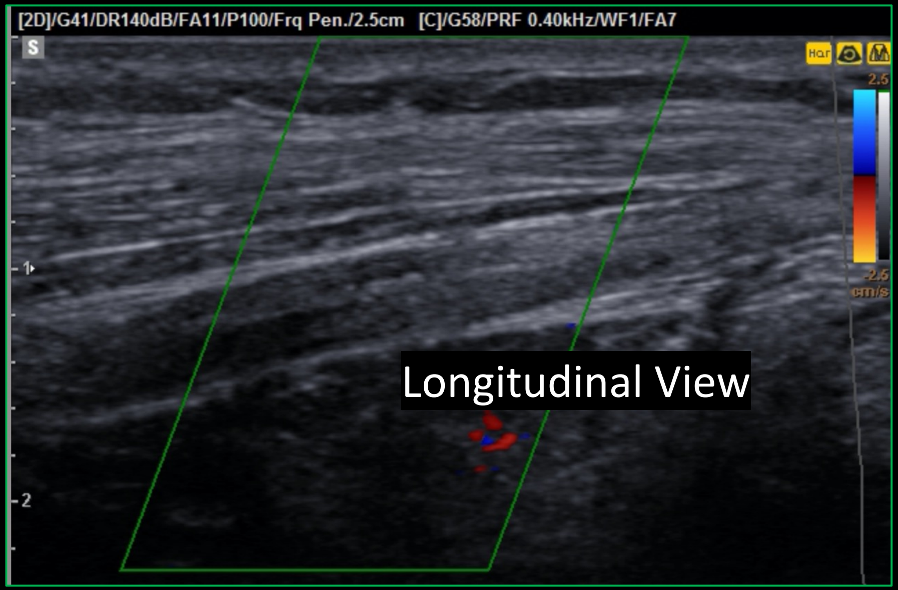

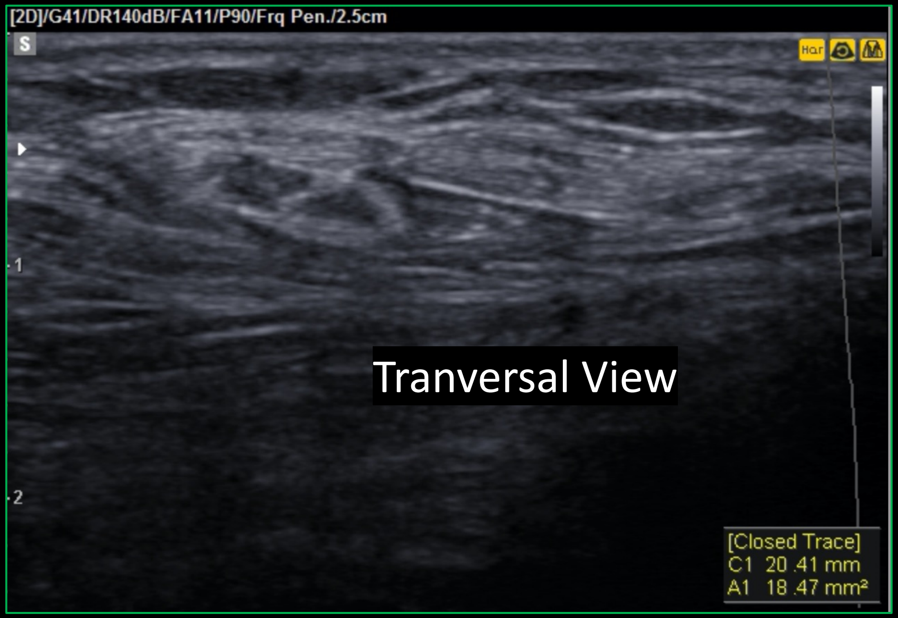

Left - Fibular commun Nerve

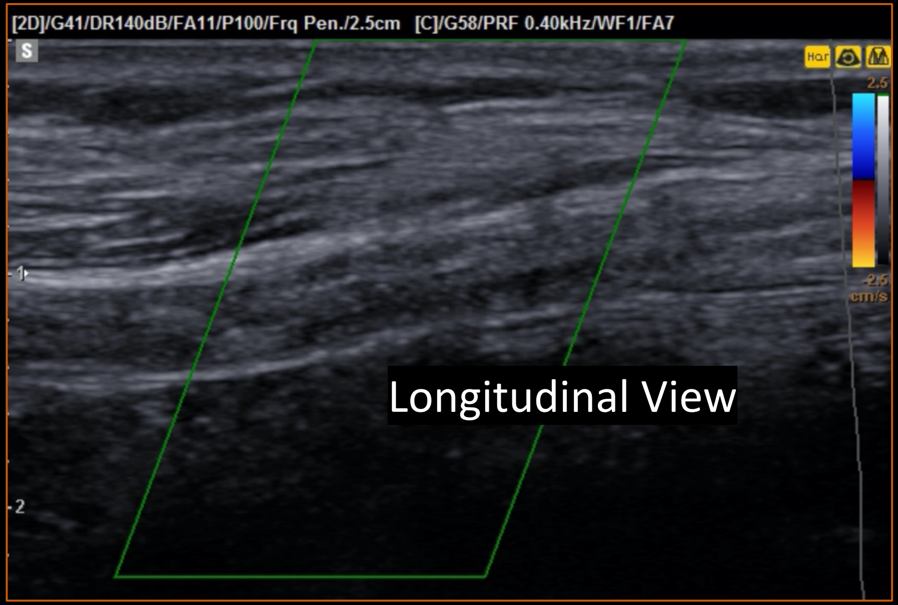

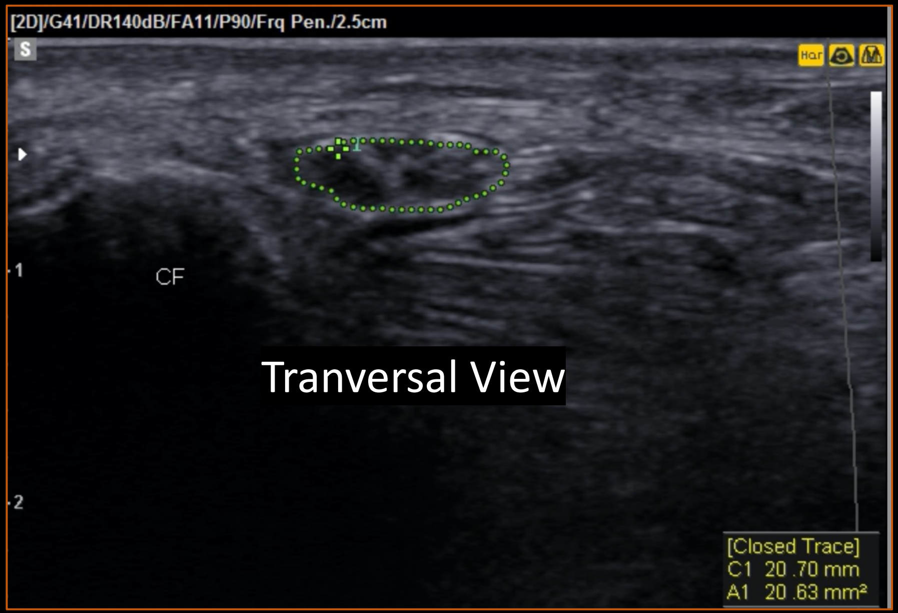

Man, 57 years, household contact of HD-patient, without clinical neuropathy.

Left and Right CSA→ > UPL (18,3 mm^2)

ΔCSA = 20,63 – 18,47 = 2,2mm^2 < ULV (2,5 mm^2)

Woman, 38years, HHC without clinical neuropathy. → HRUS report: mononeuropathy.

Fibular commun nerve with enlargement (CSA→ > UPL 18,3 mm^2) in two points (proximal of fibular head and fibular head. ΔTpt = 24,41– 22,78 = 1,63 mm^2 → < ULV (2,5 mm^2)

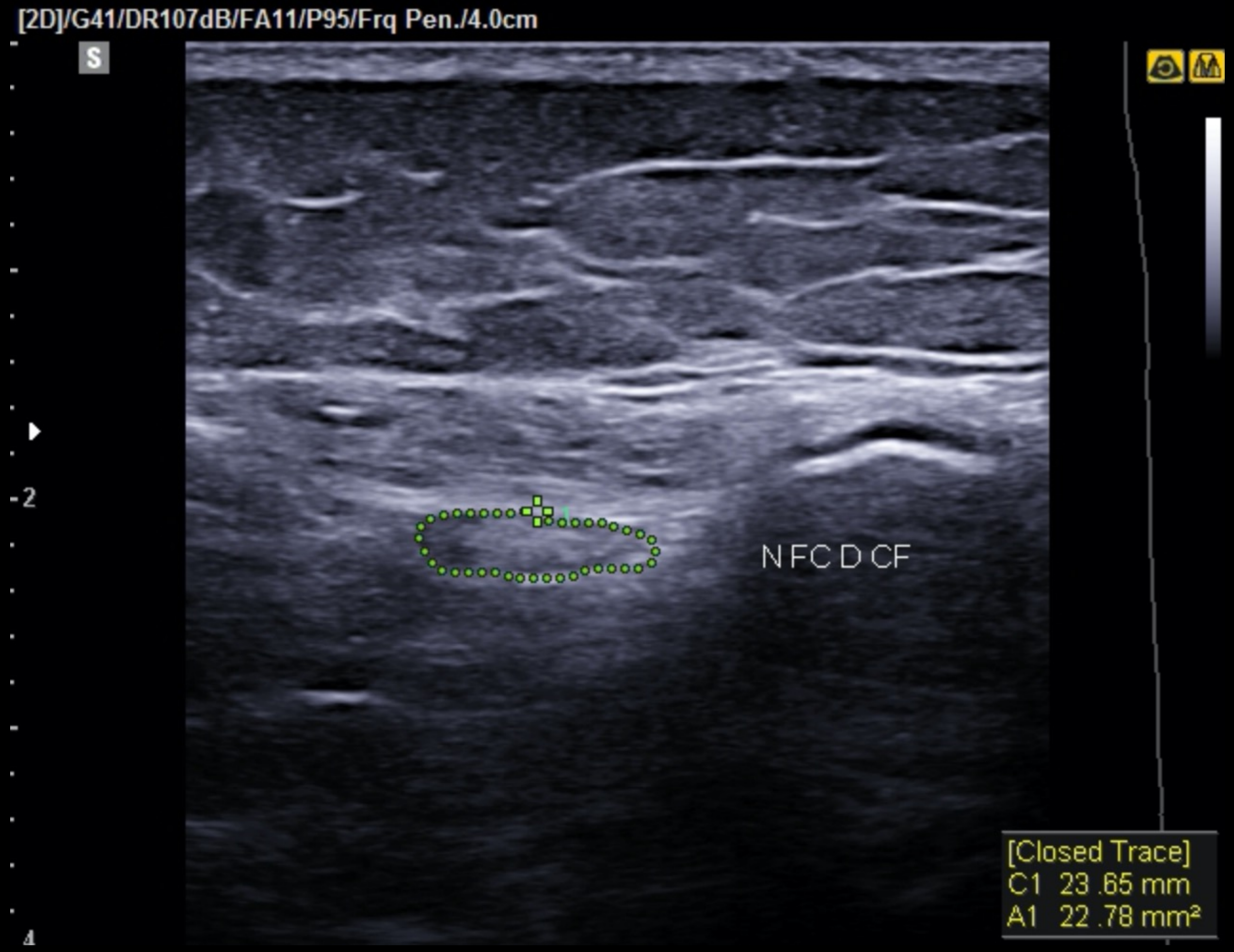

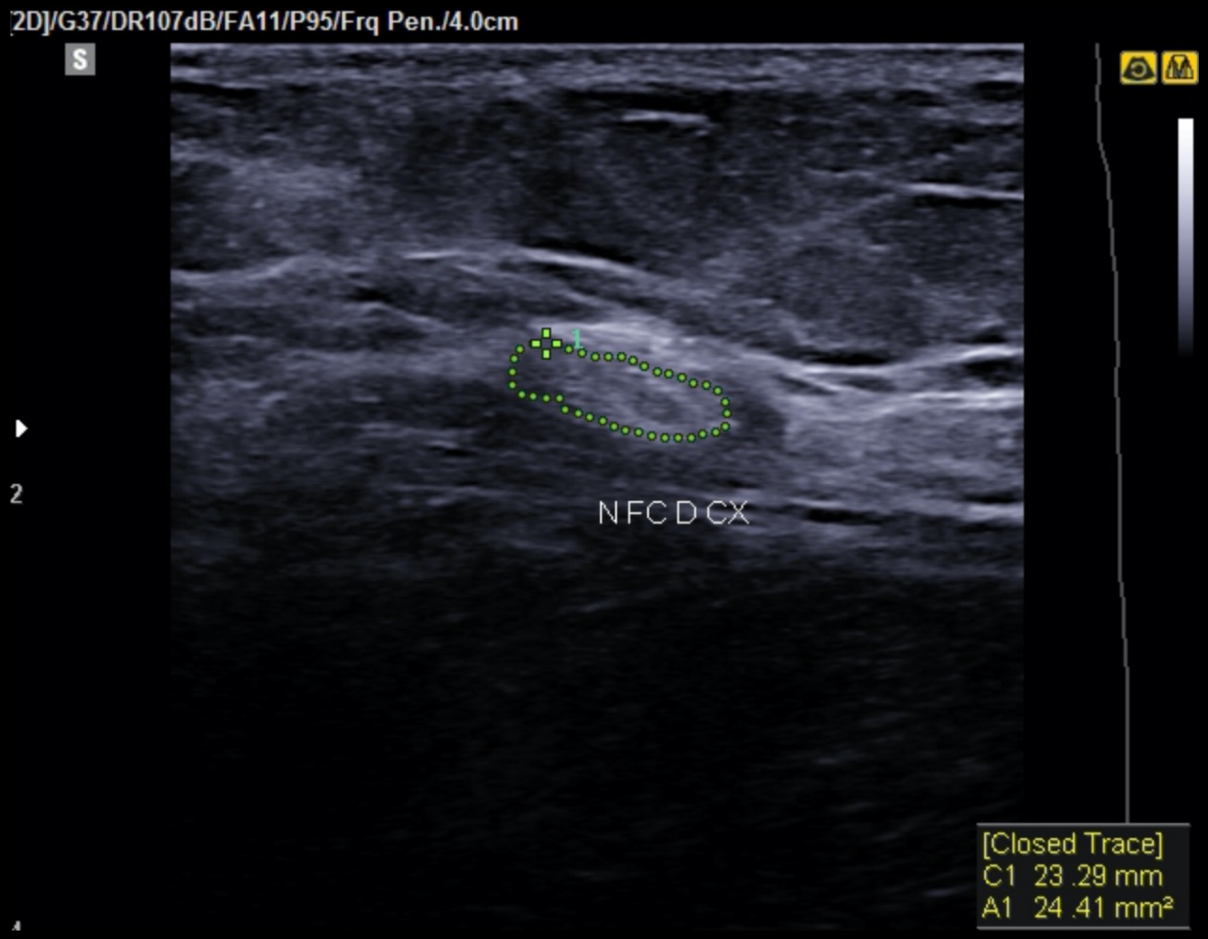

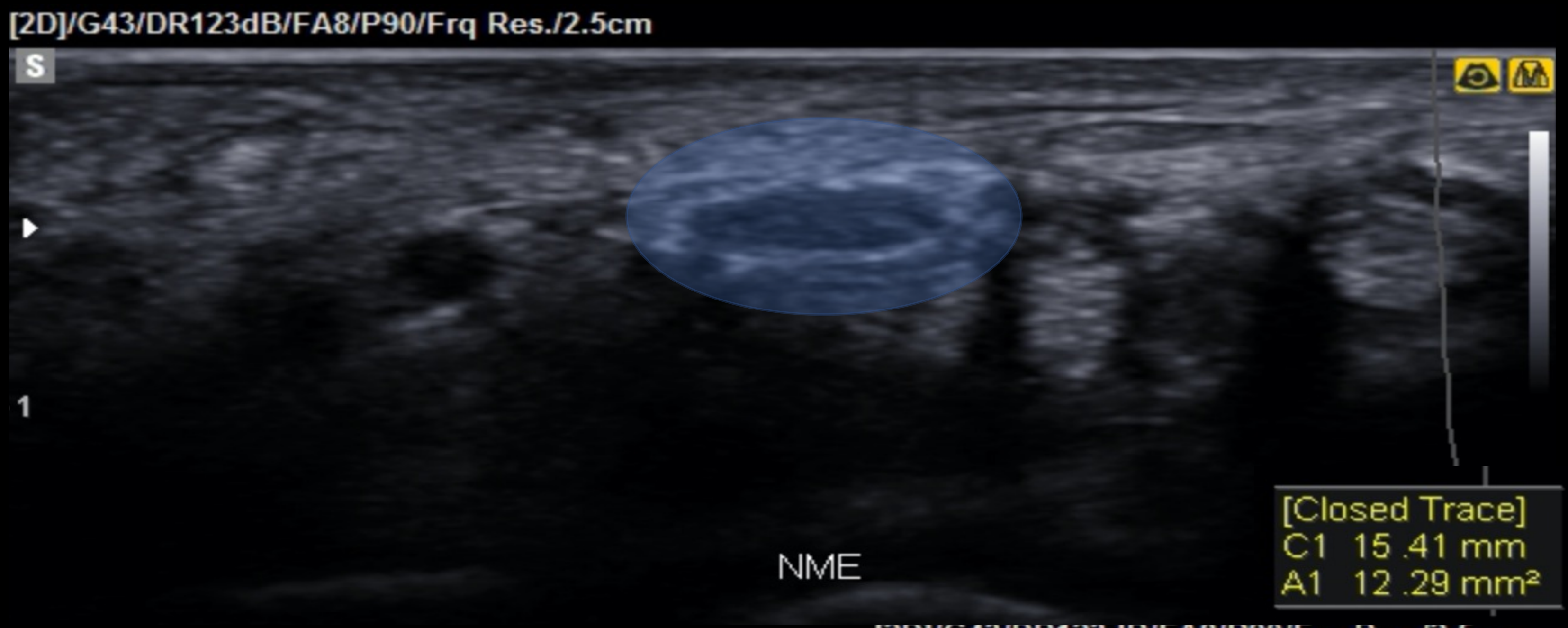

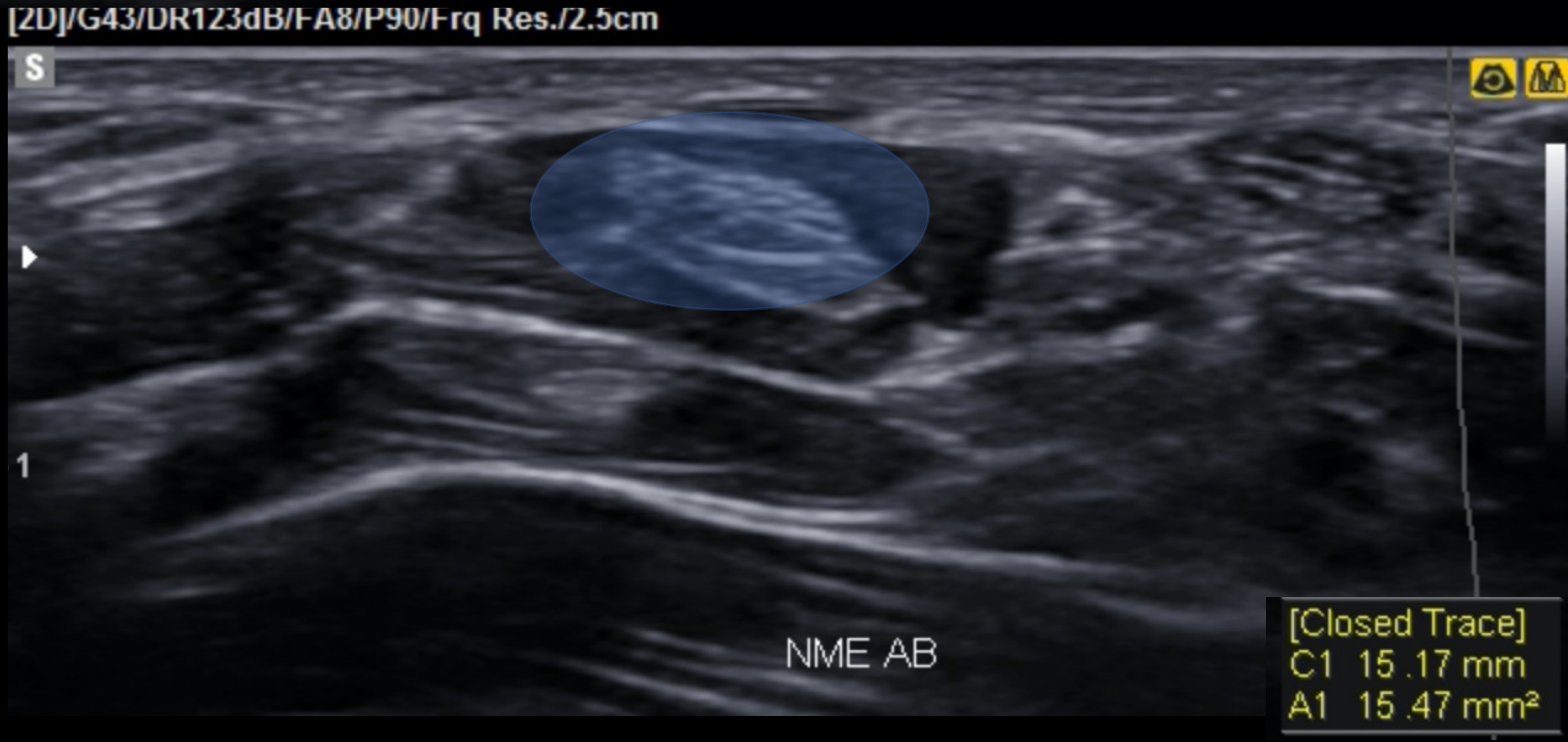

Woman with 36 years, contact of HD-patient. Fusiform enlargement of left median nerve highlight to proximal carpal tunnel with major CSA; ΔTpT = 15,47 –12,29 = 3,2 mm^2 > ULV

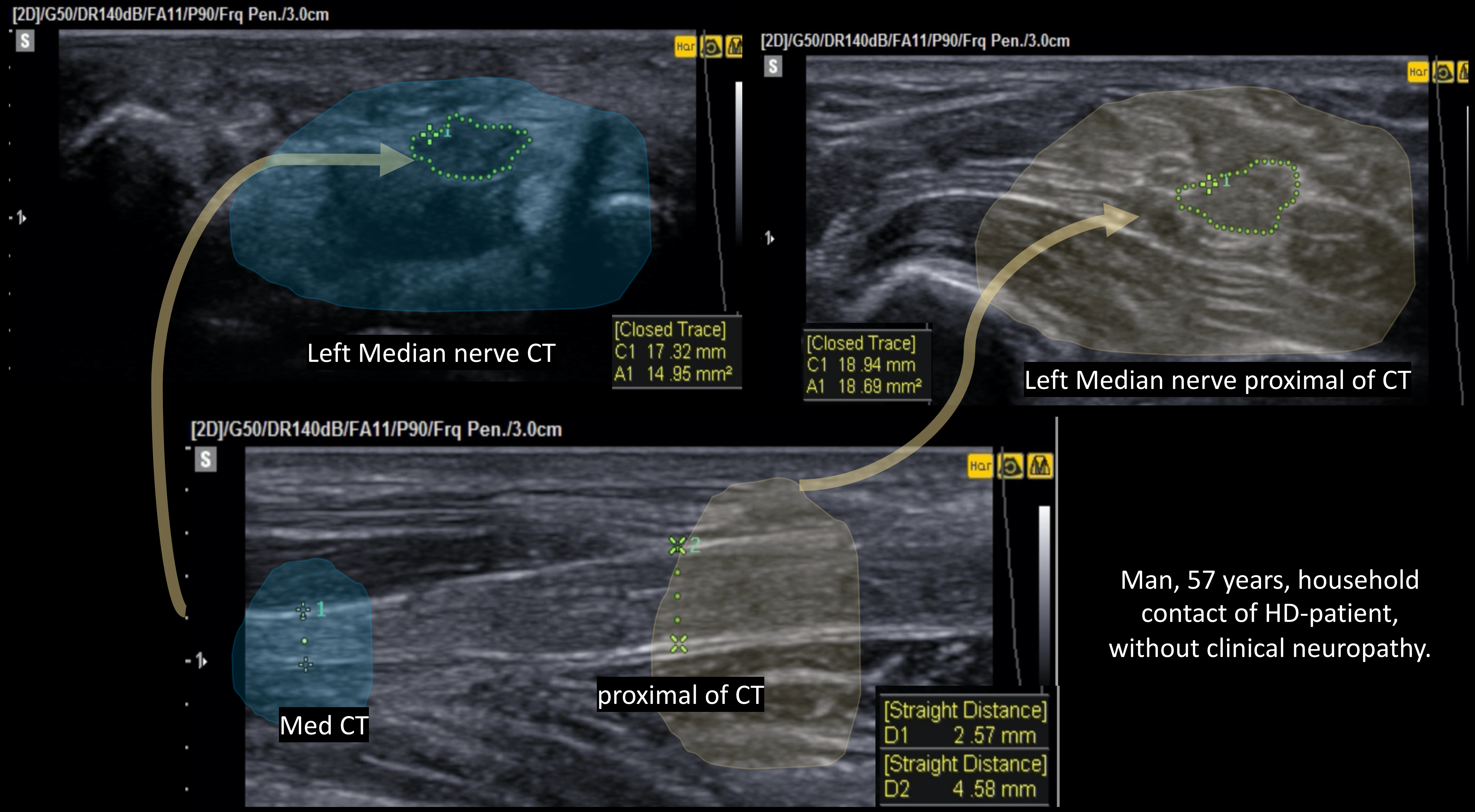

Man, 57 years, household contact of HD-patient, without clinical neuropathy.

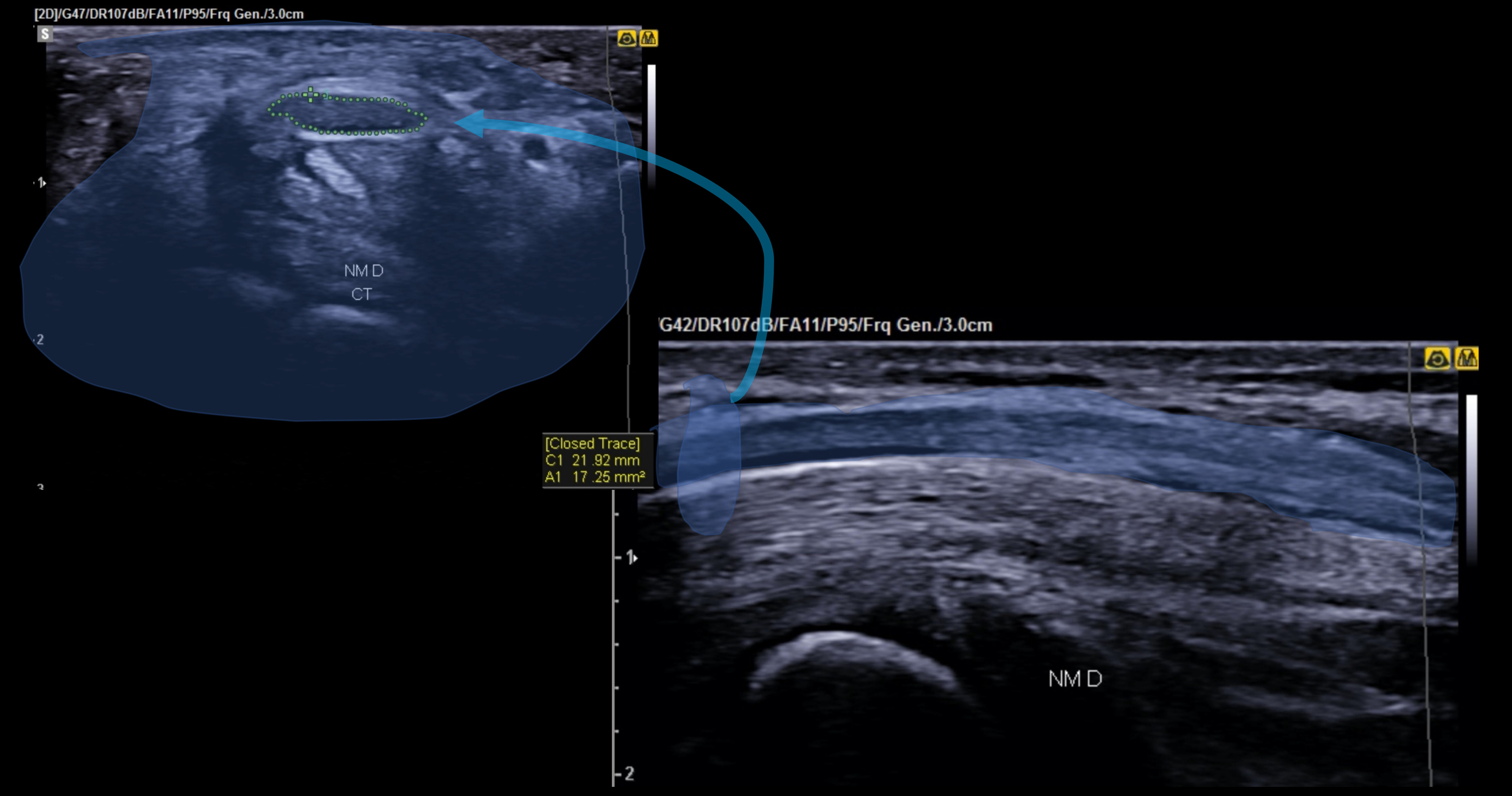

[2D]/G47/DR107dB/FA11/P95/Frq Gen./3.0cm
NM D
CT
[Closed Trace]
C1 21 .92 mm
A1 17 .25 mm²
/G42/DR107dB/FA11/P95/Frq Gen./3.0cm
NM D

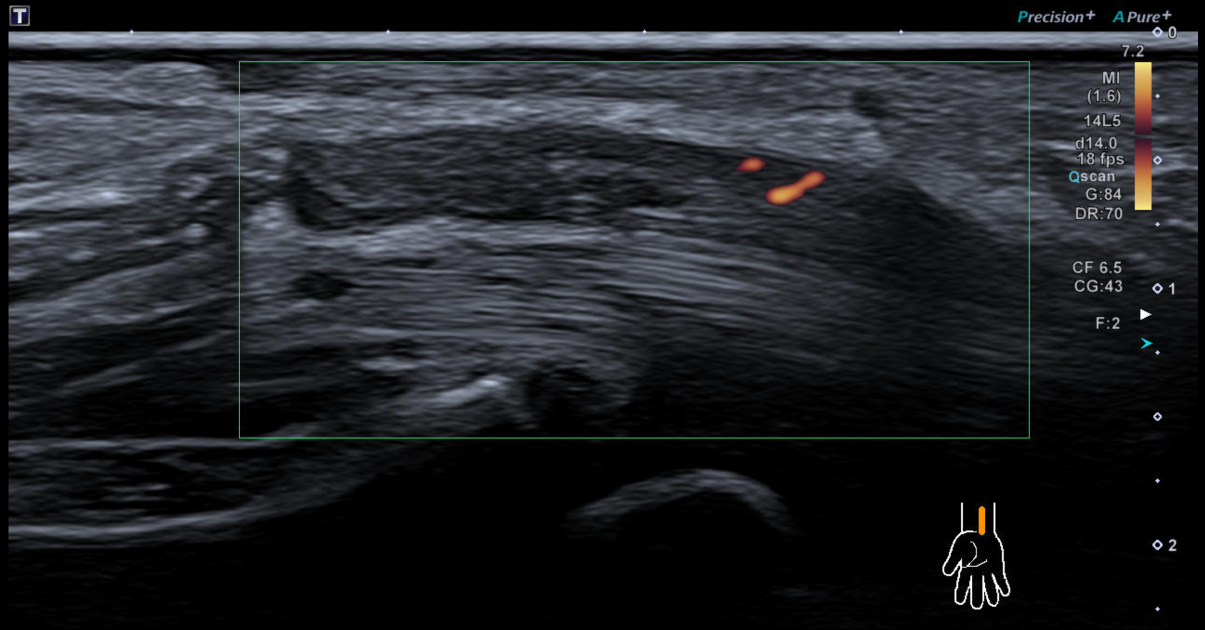
Precision+
Pure+
MI
(1.6)
14L5
d14.0
18 fps
Qscan
G:84
DR:70
CF 6.5
CG:43
F:2

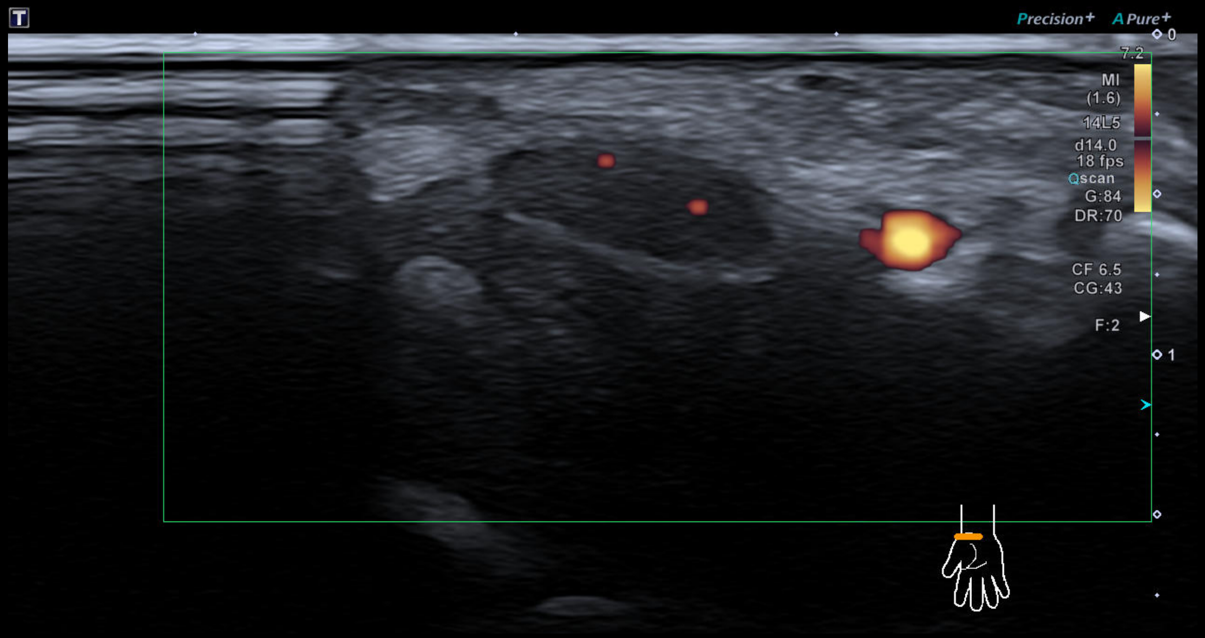
Precision+
Pure+
MI
(1.6)
14L5
d14.0
18 fps
Qscan
G:84
DR:70
CF 6.5
CG:43
F:2

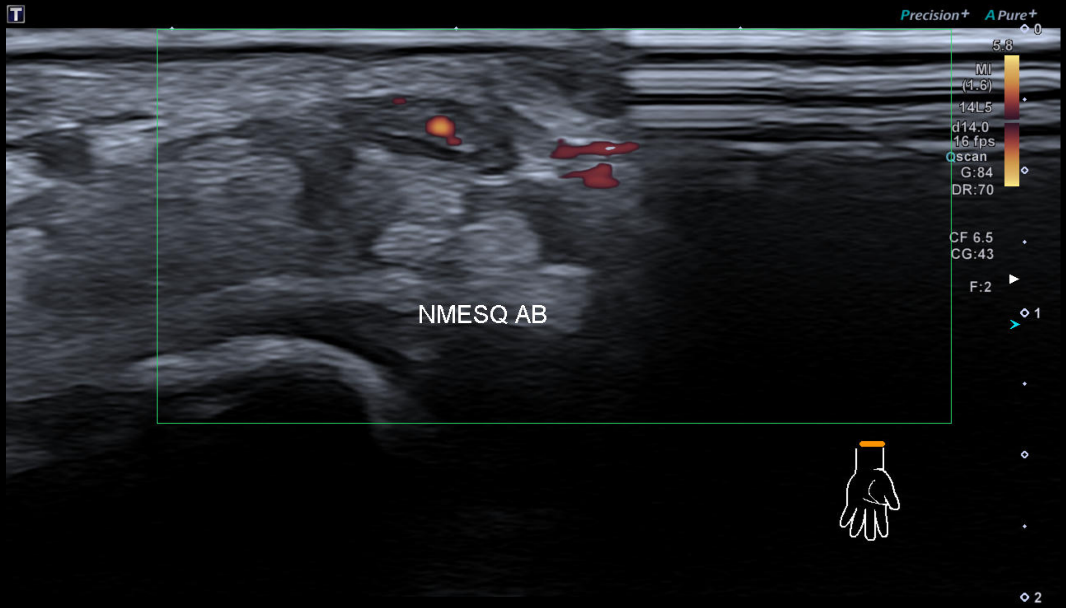
Precision+
Pure+
5.8
MI
(1.6)
14L5
d14.0
16 fps
Qscan
G:84
DR:70
CF 6.5
CG:43
F:2
NMESQ AB

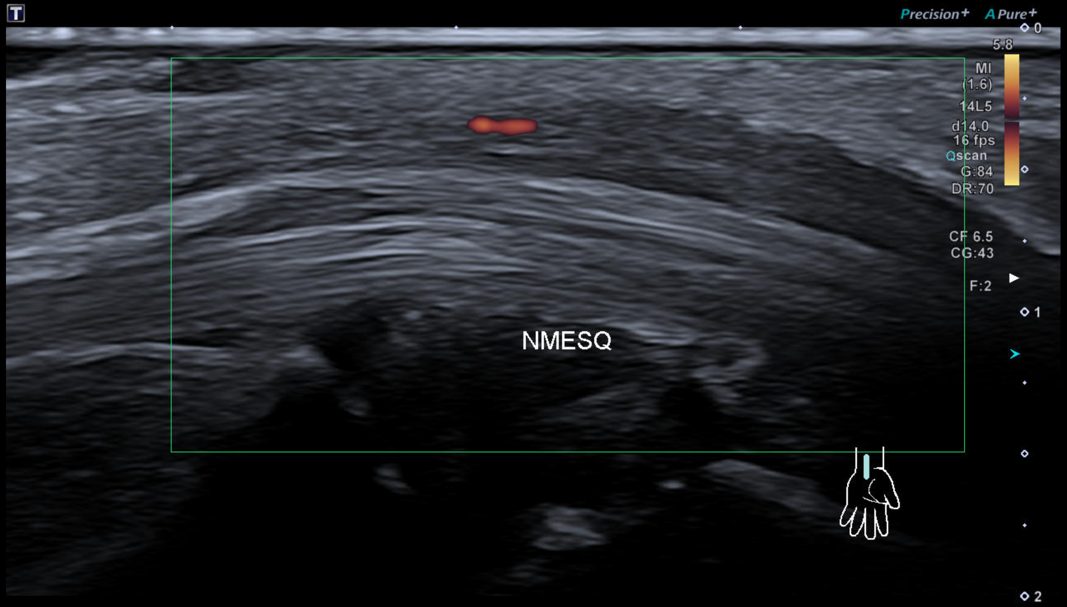
Precision+
Pure+
5.8
MI
(1.6)
14L5
d14.0
16 fps
Qscan
G:84
DR:70
CF 6.5
CG:43
F:2
NMESQ

Pattern B or Thickened CSA > VR + 2 SD

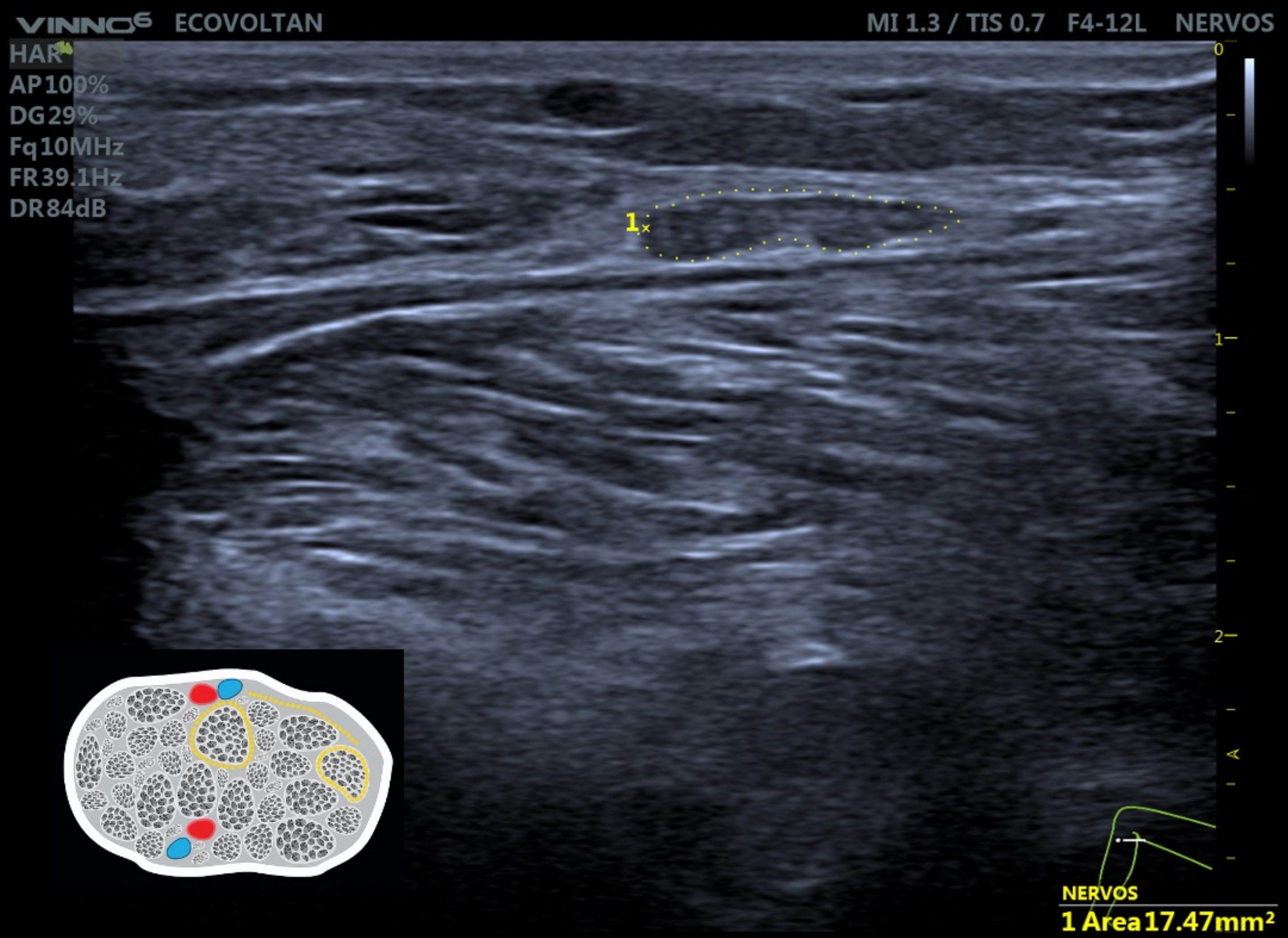

Pattern C or Hypoechoic (CSA < VR + 2 SD)

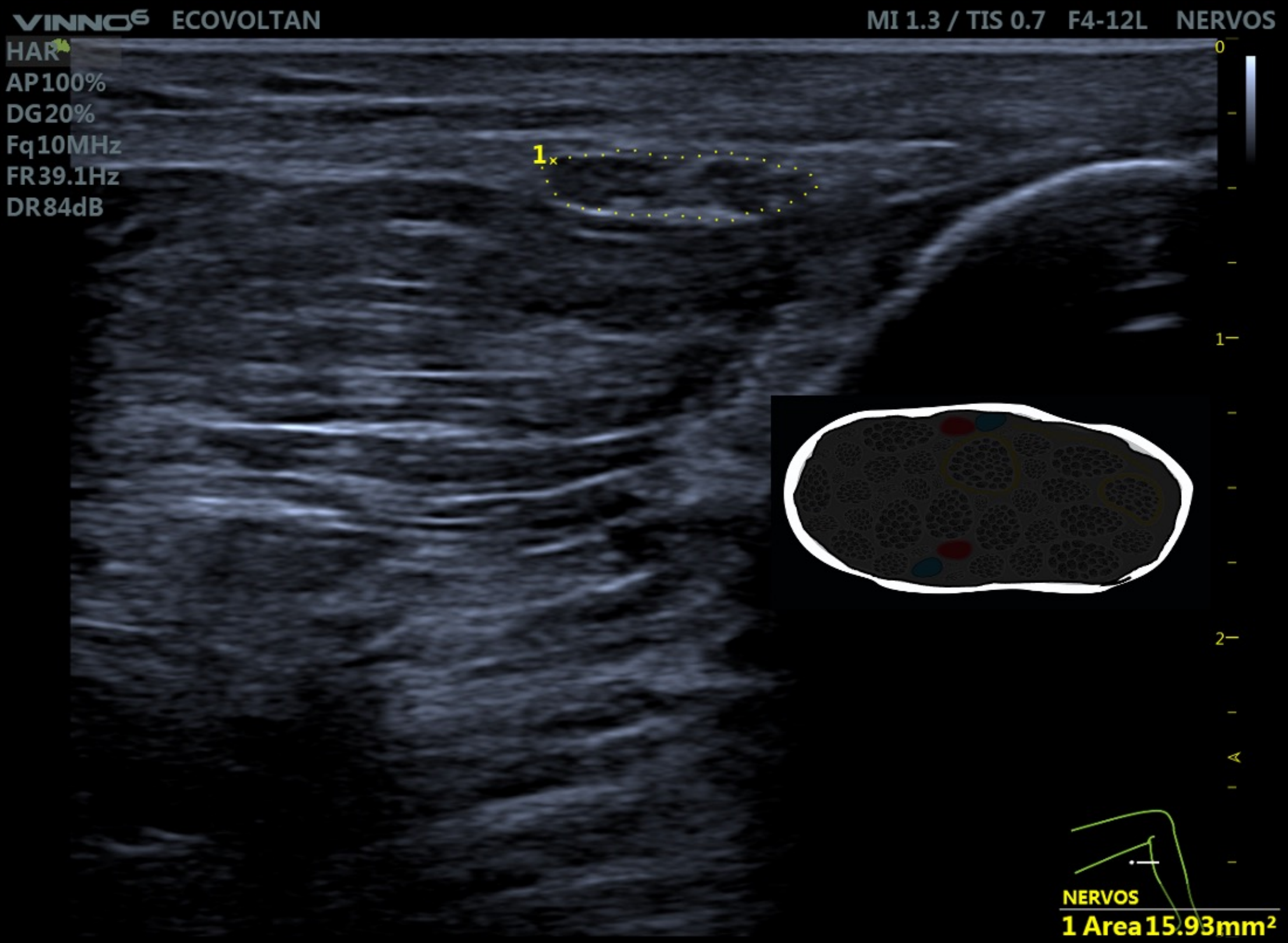

Pattern B + C or Enlargement + Hypoechoic

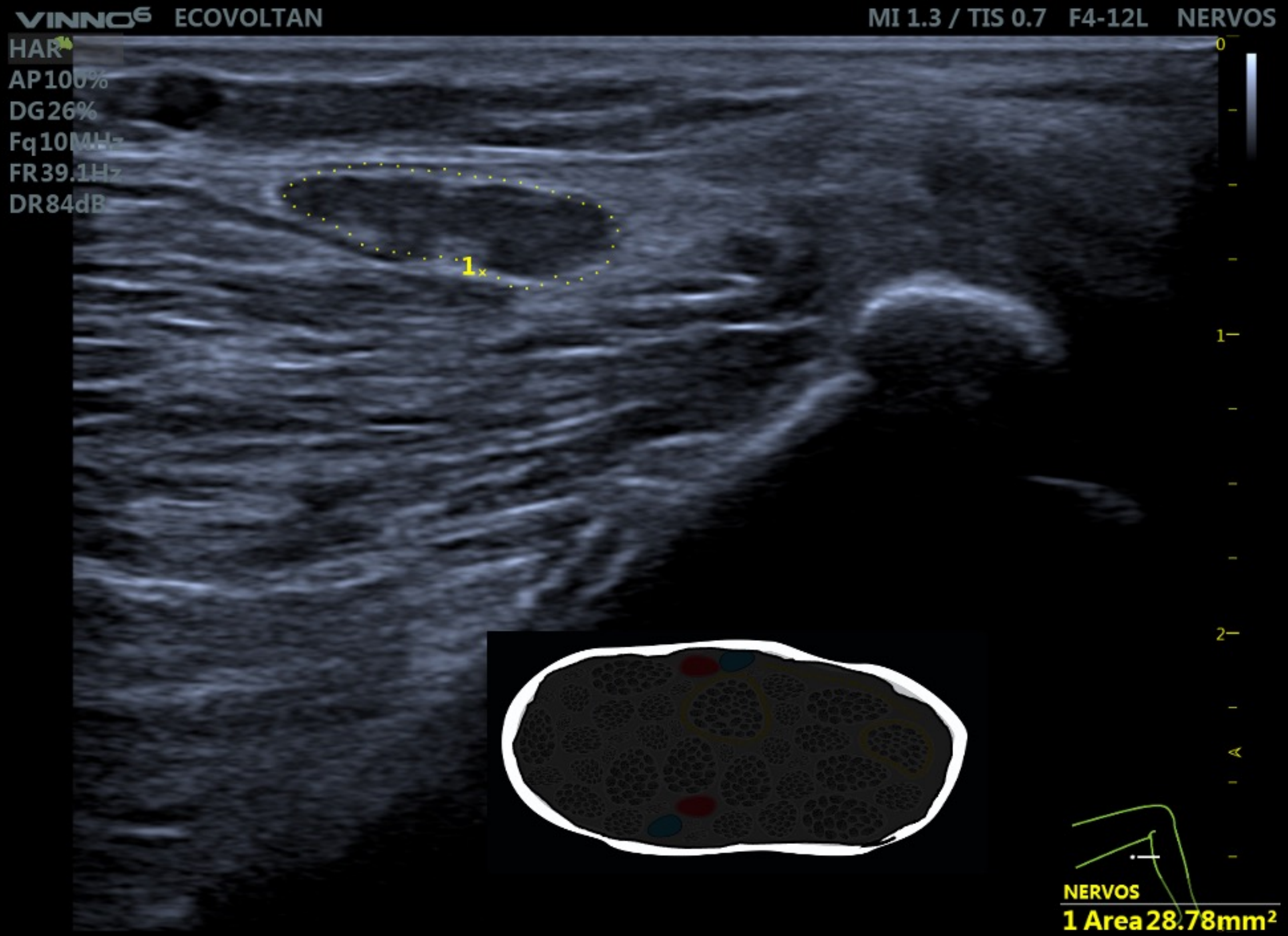

Pattern D or Focal fascicle distension (CSA < VR + 2 SD)

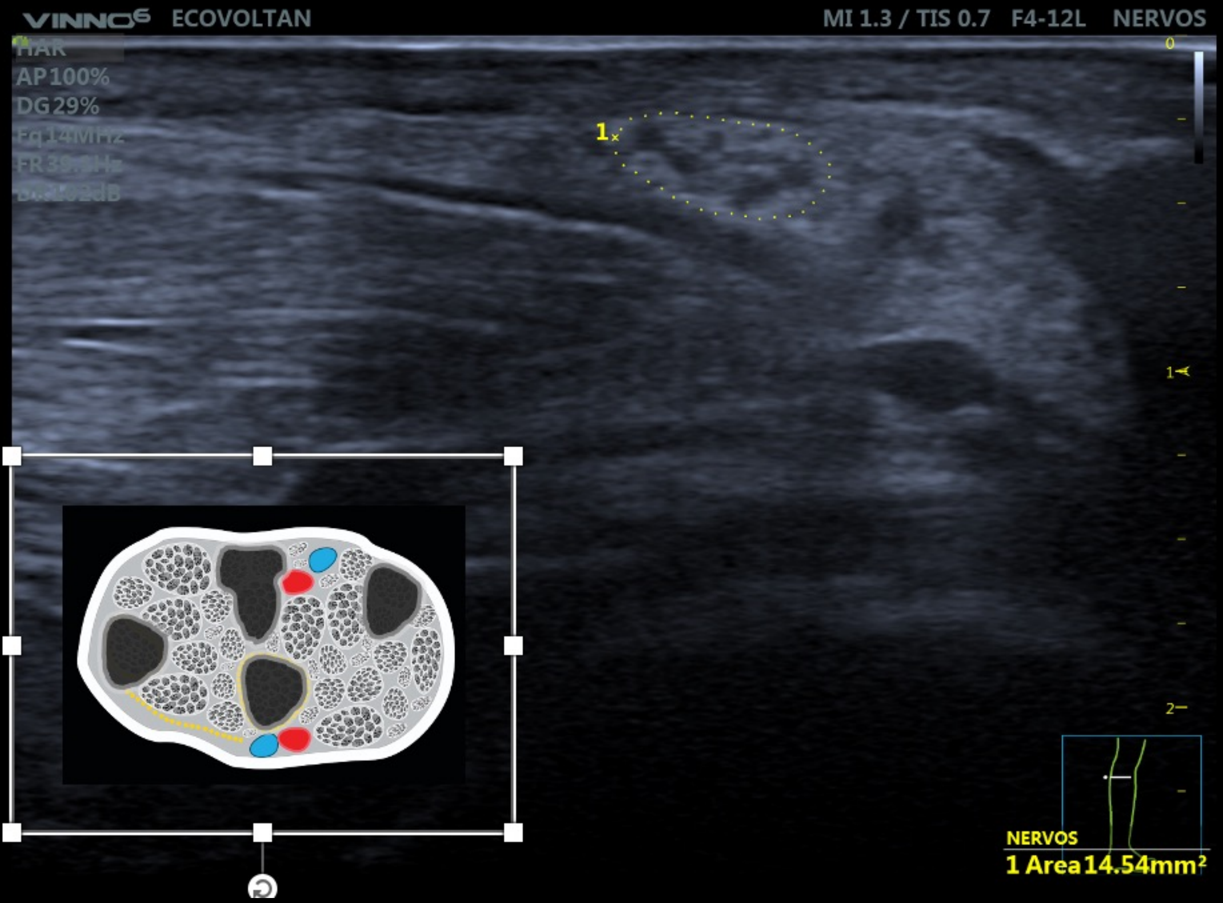

Pattern D or Focal fascicle distension (CSA < VR + 2 SD)

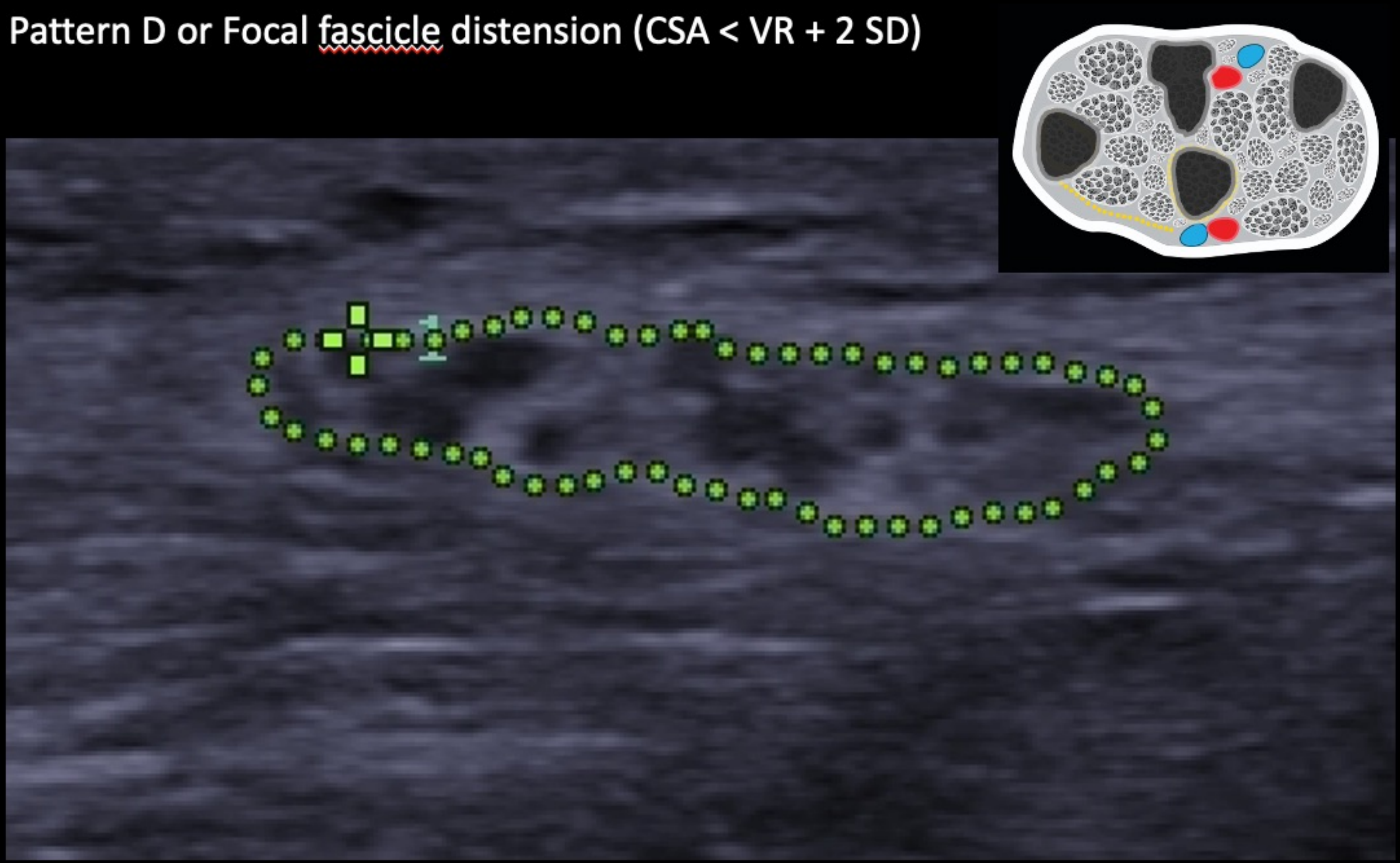

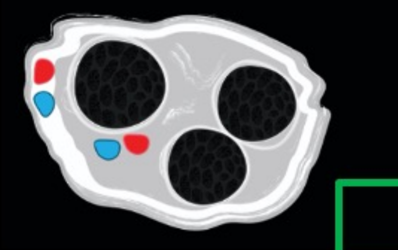

Pattern F or Heterogeneous CSA < RV + 2SD

Transversal View

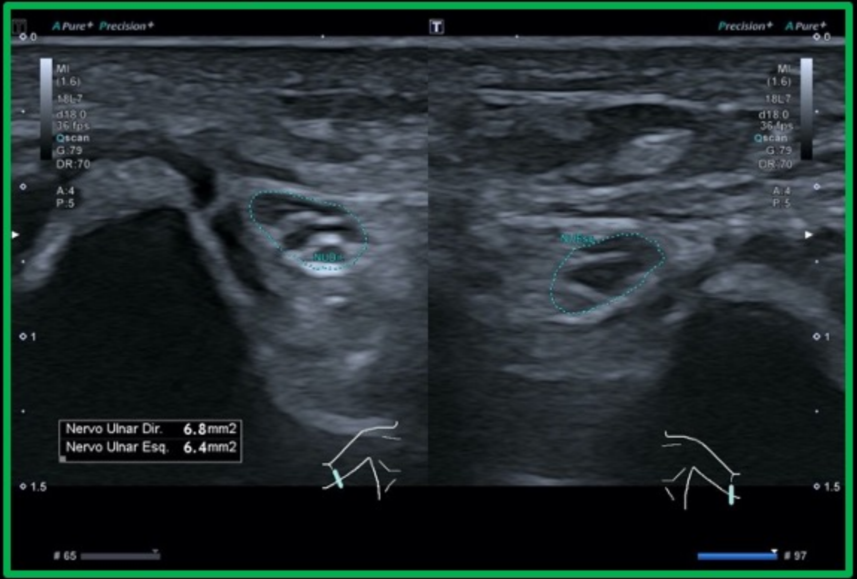

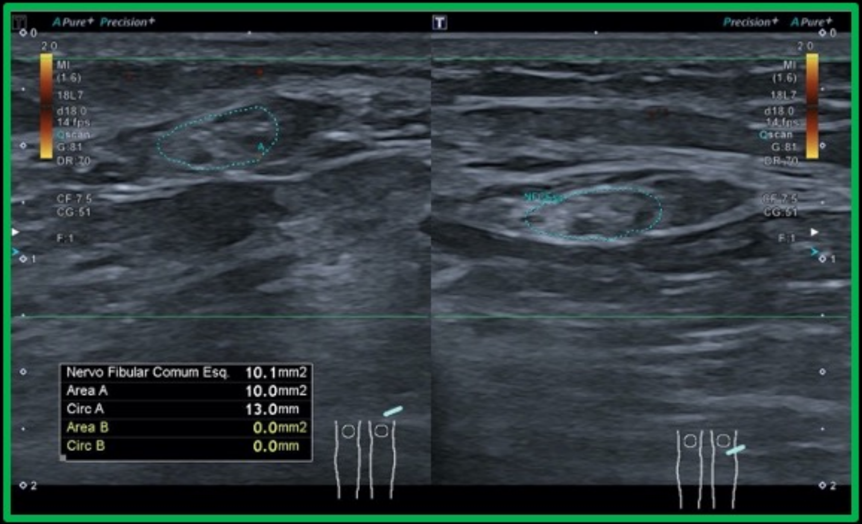

Longitudinal View

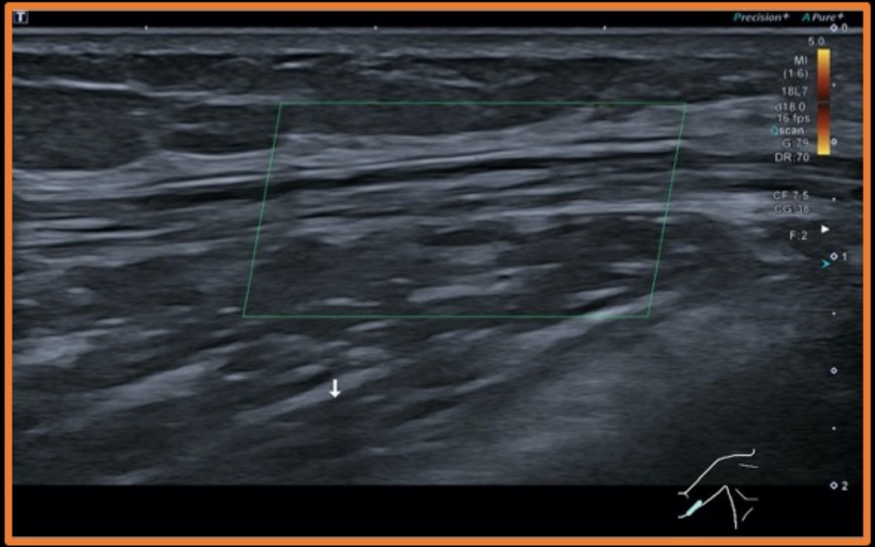

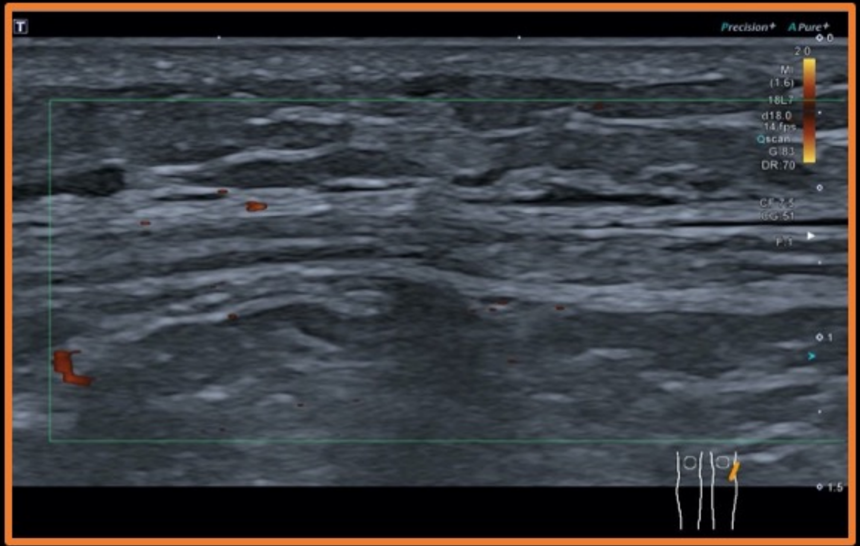

Pattern B + D or Thickened + Focal Distention

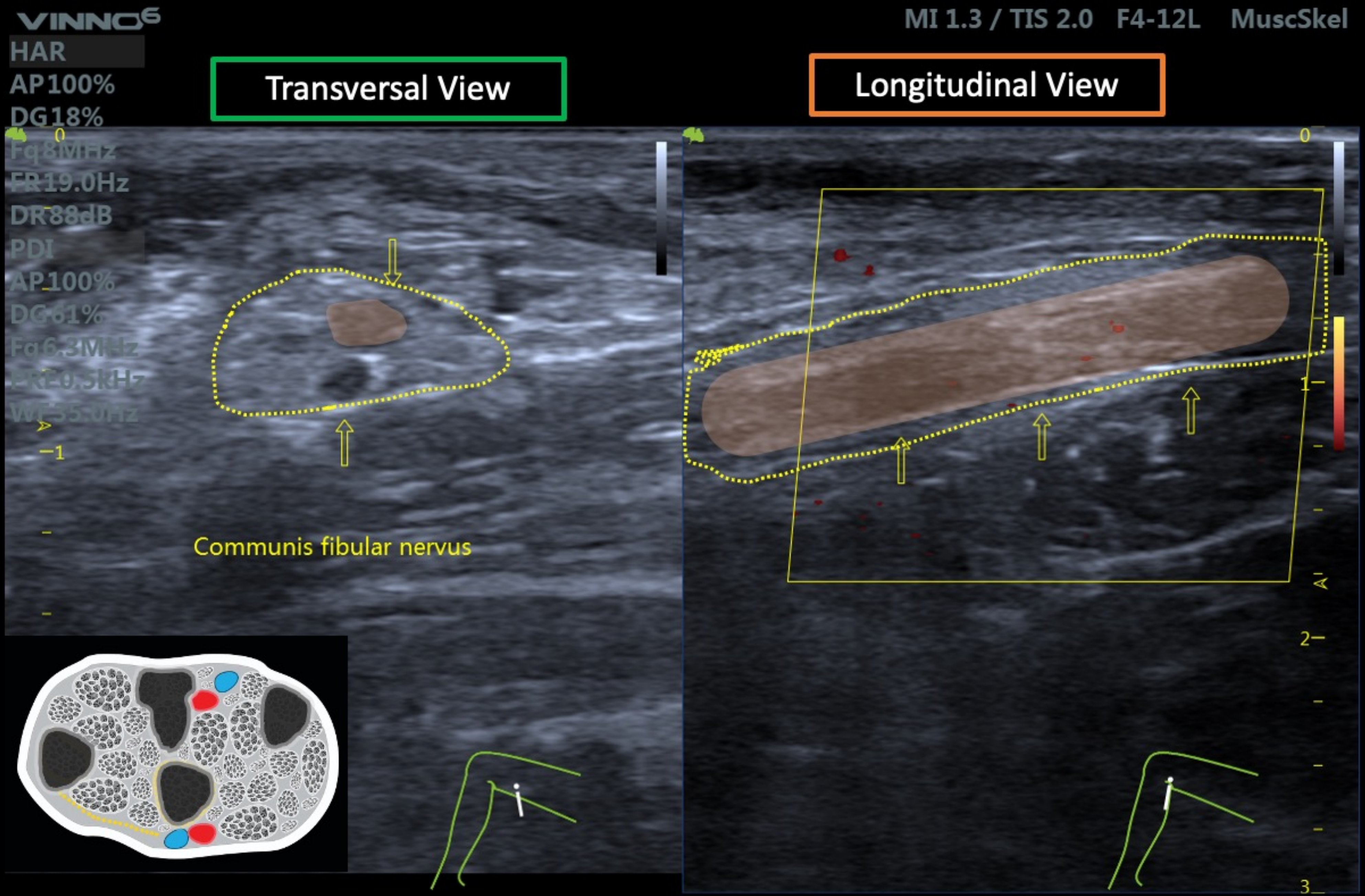

Pattern B + D + F or Thickened + Focal Distention + Heterogeneous

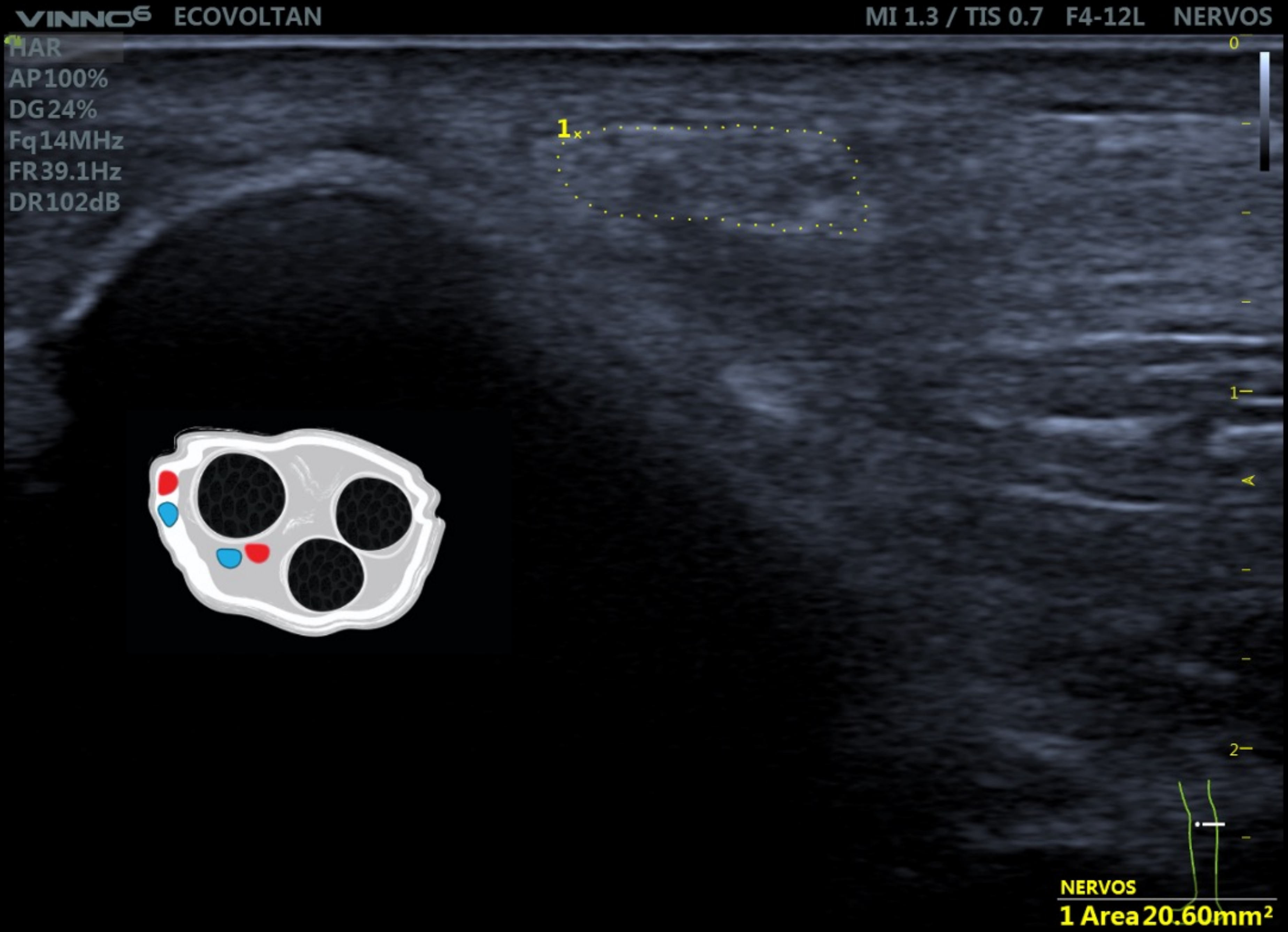

Intraneural focality (yellow): Scollard pattern modified by Voltan, G and Frade, MAC.

D pattern or Focal fascicle distention

Pattern A or Honeycombs

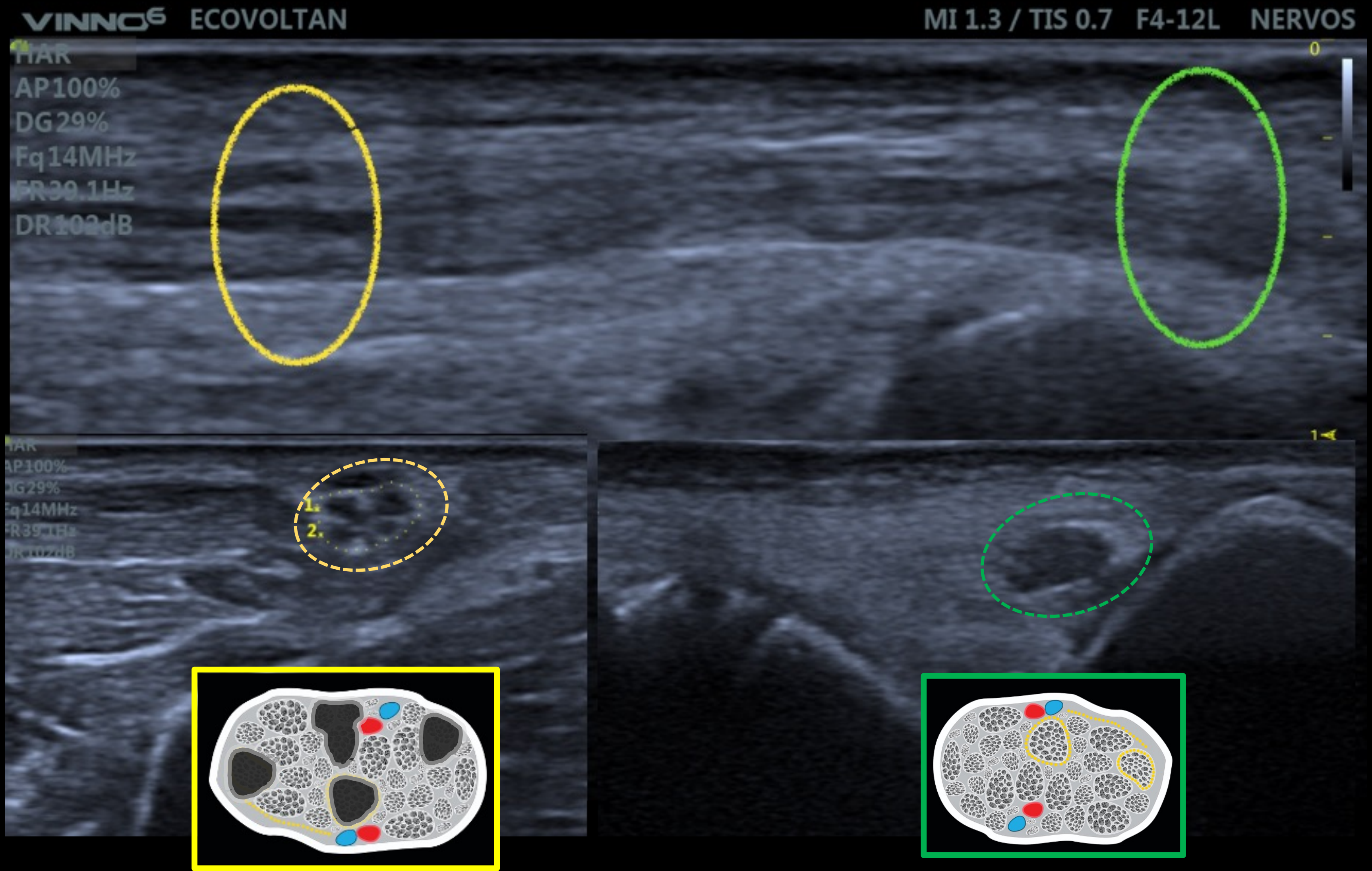

Intraneural focality (yellow): Scollard pattern modified by Voltan, G and Frade, MAC.

Pattern B or Thickened

D pattern or Focal fascicle distention

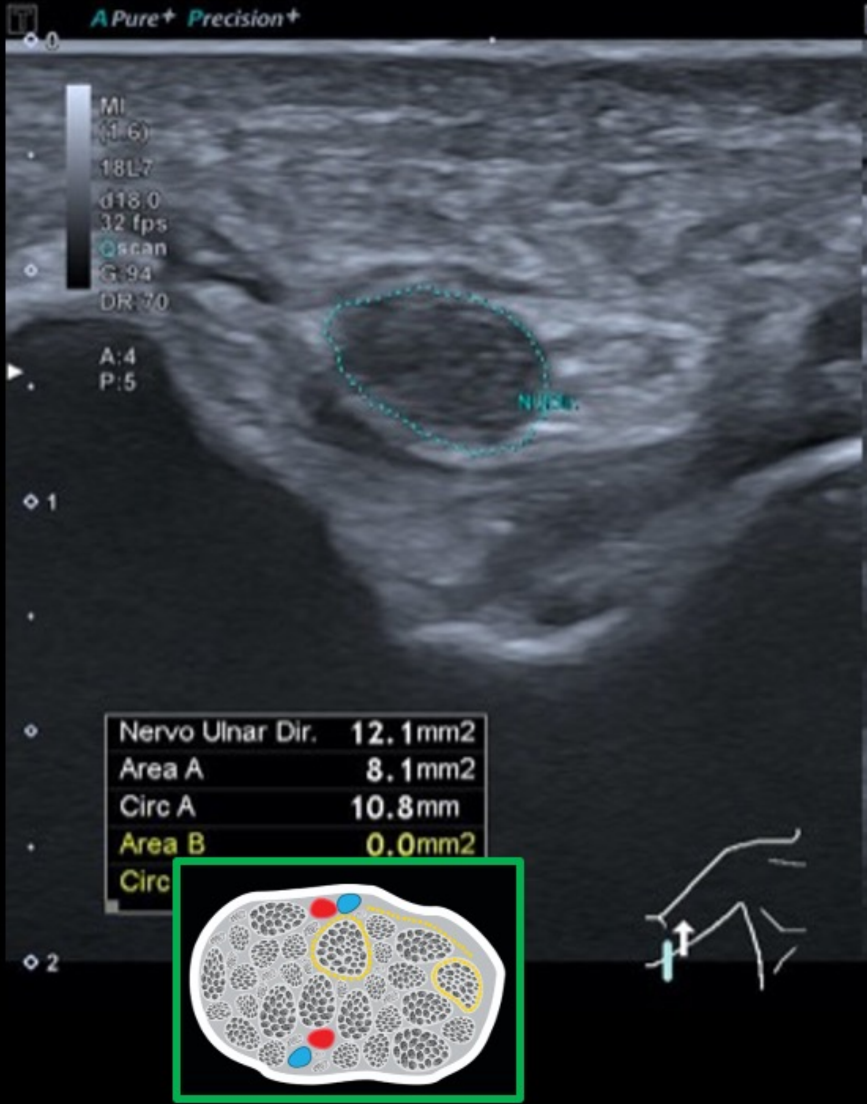

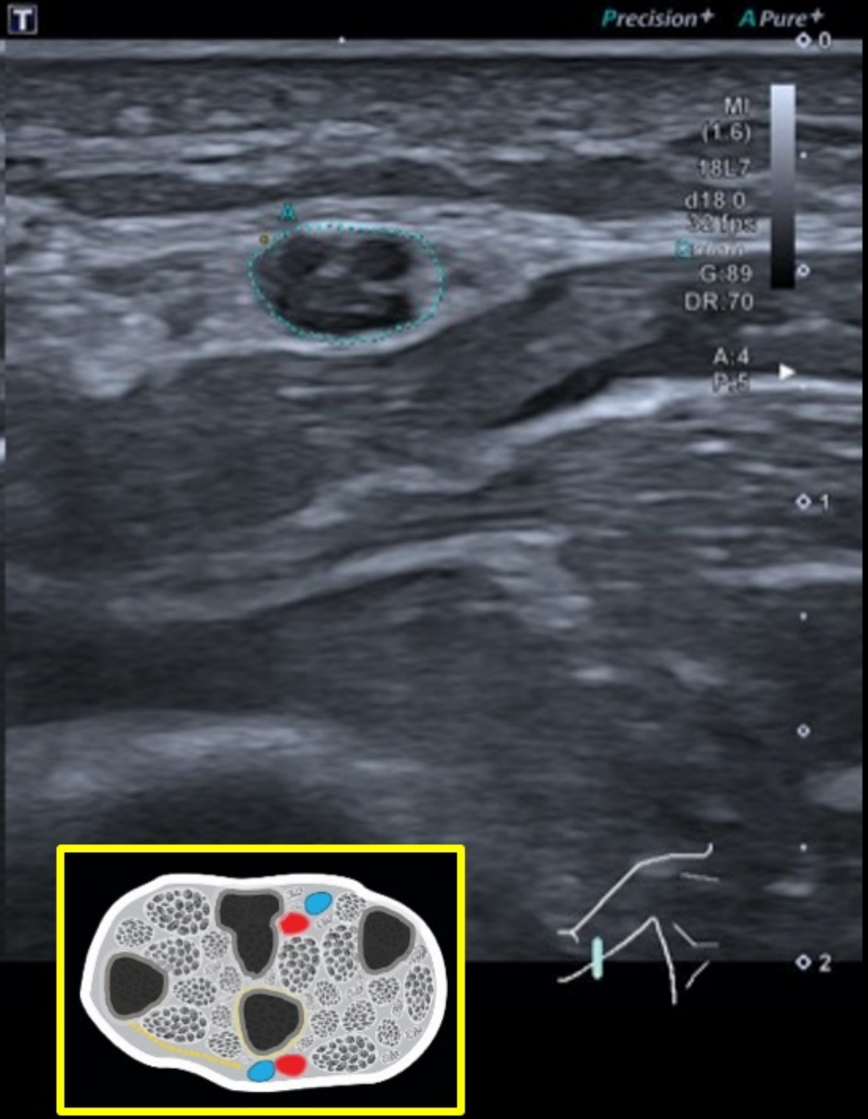

Supplement: Supplementary file 2 [file Data_Sheet_1.pdf]
